# Ruthenium Nanoparticles Stabilized with Methoxy-Functionalized Ionic Liquids: Synthesis and Structure–Performance Relations in Styrene Hydrogenation

**DOI:** 10.3390/nano13091459

**Published:** 2023-04-25

**Authors:** Deepthy Krishnan, Leonhard Schill, M. Rosa Axet, Karine Philippot, Anders Riisager

**Affiliations:** 1 Centre for Catalysis and Sustainable Chemistry, Department of Chemistry, Technical University of Denmark, DK-2800 Kgs. Lyngby, Denmark; 2 CNRS, LCC (Laboratoire de Chimie de Coordination), 205 Route de Narbonne, BP44099, CEDEX 4, 31077 Toulouse, France; rosa.axet@lcc-toulouse.fr; 3 Université de Toulouse, UPS, INPT, CEDEX 4, 31077 Toulouse, France

**Keywords:** functionalized ionic liquid, metal nanoparticle, ruthenium, catalysis, hydrogenation, styrene

## Abstract

A series of ruthenium nanoparticles (RuNPs) were synthesized by the organometallic approach in different functionalized imidazolium ionic liquids (FILs). Transmission electron microscopy (TEM) showed well-dispersed and narrow-sized RuNPs ranging from 1.3 to 2.2 nm, depending on the IL functionalization. Thermal gravimetric analysis (TGA) and X-ray photoelectron spectroscopy (XPS) allowed the interaction between the RuNPs and the ILs to be studied. The RuNPs stabilized by methoxy-based FILs (MEM and MME) displayed a good balance between catalytic activity and stability when evaluated in the hydrogenation of styrene (S) under mild reaction conditions. Moreover, the catalysts showed total selectivity towards ethylbenzene (EB) under milder reaction conditions (5 bar, 30 °C) than reported in the literature for other RuNP catalysts.

## 1. Introduction

Ionic liquids (ILs) are entropic drivers for the spontaneous self-assembly of nanoscale structures due to steric, electrostatic and stabilization properties [1]. Accordingly, they are attractive media for the synthesis of metal nanoparticles (MNPs), where they can further act as a solvent for their growth [2]. Tuning the properties of ILs, such as changing the alkyl chain length of the imidazolium cation [3], incorporating a coordinating group [4] or varying the ion pair [5,6], as non-exhaustive examples, can influence the synthesis of MNPs, and consequently, their structural and surface properties. For any applications with MNPs, the tuning of structural and surface properties can be a way to adjust their performance. This is particularly interesting in the domain of catalysis where IL-stabilized MNPs have attracted increased attention [7,8], including Ru-based systems [9]. The weakly bound IL stabilizer can easily be displaced from the NP surface, thus allowing the exposure of free and active metal sites for the catalysis. However, this ability of ILs can also be a limitation as it can result in aggregation of the MNPs during catalysis. Hence, to provide an effective balance between the stability and activity of MNPs, an alternative is to use functionalized ionic liquids (FILs) or task-specific ionic liquids (TSILs) as stabilizers [10]. In FILs, the functional group can be incorporated into either the anion or the cation of the IL [11]. Several examples of MNPs stabilized by imidazolium-based ILs containing carboxylic acid- [12], phosphine- [13], cyano- [14], ether- [15] and hydroxy- [16] functional groups have been reported. Given the possibility of interacting with the metal surface, as conventional ligands do [17], such functionalities exhibit extra stabilization to the MNPs in addition to the usual steric and electrostatic ones [18]. Hence, the modification of the FILs can be an efficient strategy to improve the catalytic performance of MNPs [10].

Decomposition of organometallic complexes such as (η^4^-1,5-cyclooctadiene)(η^6^-1,3,5-cyclooctatriene)ruthenium(0), [Ru(COD)(COT)], with H_2_ under mild conditions is a powerful method to access small and well-controlled ruthenium NPs (RuNPs) for application in catalysis [19]. When using an organic solvent and a convenient stabilizer (polymer or ligand), the organometallic approach allows control of the surface properties of the RuNPs. This strategy has proven to also be efficient using ILs as reaction media for the formation of finely controlled RuNPs. For instance, Santini and coworkers found, for a series of alkyl-substituted imidazolium bis(trifluoromethanesulfonyl)imide ILs, namely [RMIm][NTf_2_] (M = methyl, R = ethyl, butyl, hexyl, octyl and decyl), [R_2_Im][NTf_2_] (R = butyl) and [BMMIm][NTf_2_] (B = butyl, M = methyl), that the sizes of the formed RuNPs correlated with the solubility of [Ru(COD)(COT)] in the non-polar domains of the ILs, which increased with the alkyl chain length [3]. The same group also reported the synthesis of RuNPs of various sizes (1–3 nm) from [Ru(COD)(COT)] in [BMIm][NTf_2_] IL by varying the experimental conditions. The relationship between the size and catalytic performance of RuNPs was probed by the catalytic hydrogenation of 1,3-cyclohexadiene (CYD) and cyclohexene (CYE), showing an increase in the catalytic activity with the NP size. Regarding the selectivity for CYE vs. cyclohexane (CYA), it decreased with larger RuNPs [20]. The group of Moores studied the dependency of catalytic stability and activity for RuNPs stabilized in phosphonium- and imidazolium-type ILs [P_4,4,4,1_][NTf_2_], [P_4,4,4,8_][NTf_2_] and [P_4,4,4,14_]X (X = NTf_2_, OTf, PF_6_), [BMIm][NTf_2_] and [BMMIm][NTf_2_] with respect to the ionicity of the ILs. They showed that the most stable RuNPs were formed in the ILs with lower ionicity (presence of strong associations between the IL cation and anions). The IL-stabilized RuNPs were investigated in the biphasic hydrogenation of CYE to CYA, highlighting that the stability and activity of the NPs depended on the nature of the IL [21]. Starting from another organometallic precursor, bis(2-methallyl)(1,5-cyclooctadiene)ruthenium(0), [Ru(COD)(2-methylallyl)_2_], Dupont and coworkers reported the synthesis of RuNPs in the cyano-FIL [(CH_3_CH_2_CH_2_CH_2_CN)MIm][NTf_2_]. Their results clearly showed the influence of the FILs on the catalytic properties of the so-obtained RuNPs, which were selective towards nitrile hydrogenation compared to arene hydrogenation, most likely because of an interaction of the IL-nitrile functionality with the Ru surface that prevented arene hydrogenation [22]. Pádua and coworkers studied the solvation and stabilization mechanism of RuNPs using density functional theory (DFT) methods, demonstrating that 2 nm size RuNPs were solvated by both the anions and the cations of the ILs, where the interface layer was one ion thick [23].

Selective hydrogenation of styrene (S) into ethylbenzene (EB) is of great interest in the petroleum industry, where EB is widely used in value-added aromatics and in the gasoline pool [24]. For the selective hydrogenation of S to EB, most of the literature describes the use of catalysts such as polyethylene glycol stabilized Pd [25], carbon nanotubes (CNTs) supported Pt [26] or bimetallic systems (Ni-CeO_x_/Pd) [27], whereas very few examples are reported for S hydrogenation using RuNPs in ILs/FILs. Vignolle and coworkers synthesized RuNPs stabilized with polymerized ILs (PILs) based on *N*-vinyl imidazolium and hydrogenated S (40 °C, 15 bar H_2_) as a model reaction to study the correlation between chemo-selectivity and the nature of counter anion. They showed that I^−^/NTf_2_^−^ anion exchange enabled the chemo-selective hydrogenation of S to switch from EB to ethylcyclohexane (ECH) [28]. Jiang et al. reported the synthesis of RuNPs from RuO_2_·xH_2_O in phosphine-FILs [BMMIm]_3_[tppt] (tppt = tri(*m*-sulfonyl)triphenylphosphine) and [BMMIm][PF_6_] and demonstrated that RuNPs stabilized by the former FIL had high activity and selectivity in the hydrogenation of functionalized olefins, aromatic nitro compounds and aromatic aldehydes [29]. The same group also reported the synthesis of RuNPs from RuO_2_·xH_2_O and [Ru(COD)(2-methylallyl)_2_] in phosphine-FILs such as [BMIm][tppm] (tppm = mono(*m*-sulfonyl)triphenylphosphine) and [BMIm]_3_[tppt] and their application in the chemo-selective hydrogenation of diverse substrates such as vinylarenes, aromatic ketones, aldehydes and quinolines. In the study, S was converted selectively into EB under mild conditions (30 °C, 10 bar H_2_, S/Ru ratio of 500) with full conversion in 4 h [13]. These results paved the way for exploring the capability of RuNPs stabilized by ILs bearing functional groups other than phosphines, such as ethers, to catalyze the production of EB more efficiently and under even milder reaction conditions.

Herein, we report the synthesis and full characterization of RuNPs stabilized by two ether-FILs, 1-methoxyethoxymethyl-3-methylimidazolium bis(trifluoromethanesulfonyl) imide, [MEMIm][NTf_2_] and 1-methoxymethoxyethyl-3-methylimidazolium bis(trifluoromethanesulfonyl) imide, [MMEIm][NTf_2_], following the organometallic approach. The novel RuNPs/FIL systems, namely Ru/MEM and Ru/MME, were applied for the hydrogenation of S to probe the surface reactivity of the RuNPs. In particular, the influence of the ether functions on the catalytic performance (conversion, selectivity) was studied and compared to two counterpart catalytic systems based on RuNPs stabilized by the non-functionalized IL 1-hexyl-3-methylimidazolium bis(trifluoromethanesulfonyl)imide, [HIm][NTf_2_], (Ru/H) and the cyano-FIL 1-(4-cyanobutyl)-3-methylimidazolium bis(trifluoromethanesulfonyl)imide, [C_4_CNIm][NTf_2_] (Ru/CN), respectively. The obtained results indicated a clear influence of the FIL on the hydrogenation selectivity of the RuNPs between vinyl and aromatic hydrogenation of S, thus demonstrating the key role of the FIL in the catalysts. 

## 2. Materials and Methods

### 2.1. General Methods

All operations for the synthesis of RuNPs and the preparation of the catalytic tests were performed under inert atmosphere (argon) using standard Schlenk techniques or in a glovebox (MBraun). Pentane, acetonitrile, acetone, dichloromethane (DCM) and diethyl ether were purified by standard methods or using a MBraun SPS-800 solvent purification system, and further degassed using the freeze-pump-thaw method or argon bubbling. 1-Methylimidazole (99%, Sigma Aldrich, St. Louis, MO, USA), 1-chlorohexane (>99%, Sigma Aldrich), 1-bromo-2-methoxymethoxy ethane (98%, Sigma Aldrich), 2-methoxyethoxy methyl chloride (99%, Sigma Aldrich), 5-chlorovaleronitrile (98%, Sigma Aldrich), lithium bis(trifluoromethylsulfonyl)imide (Li[NTf_2_]; >99%, Chemodex) were used as received. Styrene (S; 98%, Sigma Aldrich), ethylbenzene (EB; 98%, Sigma Aldrich), octane (>99%, Sigma Aldrich) were purified by filtration on an alumina column, then degassed and dried before use. [Ru(COD)(COT)] complex was purchased from Nanomeps, Toulouse, France. H_2_ (99.999%) and Ar (99.99%) were purchased from Air Liquid, Taastrup, Denmark.

### 2.2. Characterization Methods

Liquid nuclear magnetic resonance (NMR) spectroscopy (^1^H, ^13^C{^1^H}, ^19^F, ^13^C dept-135) was performed on a Bruker Avance 300 instrument or a Bruker Avance 400 instrument using CDCl_3_ or DMSO-d_6_ as solvents. Attenuated total reflection infrared (ATR-IR) spectra were recorded in the range 4000–400 cm^−1^ under inert conditions on a Perkin-Elmer GX2000 spectrometer installed inside a glovebox. Metal content was determined by inductively coupled plasma optical emission spectroscopy (ICP-OES) using a Thermo Scientific ICAP 6300 instrument. Transmission electron microscopy (TEM) and high-resolution transmission electron microscopy (HRTEM) analyses (Centre de microcaractérisation Raimond Castaing, CNRS-UAR 3623, Toulouse, France) were performed on a JEOL JEM 1011 CXT electron microscope operating at 100 kV with a point resolution of 4.5 Å or a JEOL JEM 1400 operating at 120 kV with a point resolution of 2.0 Å, and on a JEOL JEM 2100F equipped with a field emission gun (FEG) operating at 200 kV with a point resolution of 2.3 Å and high-angle annular dark-field imaging scanning transmission electron microscopy (HAADF-STEM) on a JEOL JEM-ARM200F Cold FEG operating at 200 kV with a point resolution of >1.9 Å, respectively. The TEM samples were prepared by diluting a few drops of each RuNPs/IL mixture in acetonitrile and casting a drop of the solution on a carbon-coated copper grid. Size distributions and mean sizes of the NPs were determined by measurement of at least 200 individual NPs on a given grid using the software ImageJ. Thermogravimetric analyses (TGA) were carried out on a Mettler Toledo TGA/DSC 3+ instrument under a N_2_ flow. Samples were placed into an alumina crucible and then heated from 25 to 800 °C at a heating rate of 10 °C/min. DSC analyses were conducted from −120 to 40 °C with a heating rate of 10 °C/min after depositing the samples in a sealed aluminum pan and using a Netzsch DSC 3500 Sirius instrument. X-ray photoelectron spectroscopy (XPS) analyses were performed using a Thermo Scientific system at room temperature using AlKα radiation (1484.6 eV) and a spot size of 400 μm. A flood gun was used to reduce sample charging effects and the obtained spectra were further corrected by setting the C 1s binding energy at 284.8 eV. Data processing was performed using the Avantage 4.87 software. Electrospray ionization mass spectrometry (ESI-MS) analyses were performed using a DSQ II Mass Spectrometer (Thermo Fisher Scientific) equipped with electron impact (EI) and chemical ionization (INN NH3) sources analyses up to 1000 mass at low resolution under argon. Elemental analyses (EA) were acquired on a Thermo Fischer Flash EA 1112 analyzer (Department of Chemistry, University of Copenhagen). The water content in solvents and ILs was determined with a Karl Fisher Coulometer (Metrohm) (target value for use of solvents < 5 ppm).

### 2.3. Synthesis of Functionalized Ionic Liquids (FILs)

(1-Hexyl-3-methyl imidazolium bis(trifluoromethanesulfonyl)imide (H). The synthesis of H was carried out by modifying a previously described procedure [30]. 1-Chlorohexane (13.36 g, 110.70 mmol) was added dropwise to 1-methyl imidazole (8.24 g, 100.2 mmol) and the mixture stirred for 24 h at 80 °C. The solution was then washed several times with diethyl ether (50 mL) and dried in vacuo. Li[NTf_2_] (30.17 g, 104.90 mmol) dissolved in distilled water (20 mL) was then added and the mixture stirred for 24 h at room temperature. The resulting IL (lower phase) was then separated and washed several times with distilled water (≈100 mL) until no precipitate of AgCl formed when aq. AgNO_3_ was added to the washing water. Finally, remaining water was removed by stirring the IL under vacuum (≈0.05 mbar) at 60 °C overnight. Yield: 38.90 g (85%).

^1^H NMR (400 MHz, CDCl_3_): δ (ppm) 8.97 (s, 1H), 7.29 (t, 3H), 7.26 (t, 1H), 4.17 (t, 2H), 3.95 (s, 3H), 1.86 (m, 2H), 1.32 (m, 6H), 0.88 (t, 2H). ^13^C NMR {^1^H} (101 MHz, CDCl_3_): δ (ppm) 136.44, 123.71, 122.22, 118.33, 50.42, 36.56, 31.07, 30.15, 25.89, 22.42, 13.96. ^19^F NMR (376 MHz, CDCl_3_): δ (ppm) −79.01 (s, CF_3_). ESI-MS: positive, ([%]) *m*/*z* 167.1 [HIm]^+^ (100); negative, ([%]) *m*/*z* 279.9 [NTf_2_]^−^ (100). IR ν_max_/cm^−1^: 3157(w), 2924(w), 2862(w), 1346(s), 1180(s), 1050(s). Elemental analysis (%); calculated for C_12_H_19_N_3_O_4_S_2_F_6_: C, 32.21; H, 4.28; N, 9.39; found: C, 32.01; H, 4.22; N, 9.35.

1-Methoxyethoxymethyl-3-methyl imidazolium bis(trifluoromethanesulfonyl)imide (MEM). The synthesis of MEM was achieved through a similar route as previously described [31]. 2-Methoxyethoxymethyl chloride (6.10 g, 49.00 mmol) was added dropwise to 1-methyl imidazole (4.12 g, 50.10 mmol) dissolved in dry DCM (70 mL) in a Schlenk tube under inert atmosphere. After stirring at room temperature for 24 h, the DCM was removed under vacuum, and the remaining IL washed several times with diethyl ether (≈50 mL) followed by removal of excess solvent under vacuum (≈0.05 mbar). A solution of Li[NTf_2_] (14.53 g, 52.20 mmol) in distilled water (20 mL) was then added and the mixture stirred at room temperature for 24 h. The resulting IL (lower phase) was then separated and washed several times with distilled water (≈100 mL) until no precipitate of AgCl formed when aq. AgNO_3_ was added to the washing water. Finally, remaining water was removed by stirring the IL under vacuum (≈0.05 mbar) at 60 °C overnight. Yield: 20.23 g (90%).

^1^H NMR (400 MHz, CDCl_3_): δ (ppm) 8.91 (s, 1H), 7.45 (t, 1H), 7.33 (t, 1H), 5.59 (s, 2H), 3.98 (s, 3H), 3.74 (t, 2H), 3.54 (t, 2H), 3.34 (s, 3H). ^13^C NMR {^1^H} (400 MHz, CDCl_3_): δ (ppm) 136.37, 123.80, 121.32, 118.08, 79.38, 71.23, 69.70, 58.91, 36.52. ^19^F NMR (400 MHz, CDCl_3_): δ (ppm) −81.00 (s, CF_3_). ESI-MS: positive, ([%])) *m*/*z* 171.1 [MEMIm]^+^ (100); (negative, ([%])) *m*/*z* 279.9 [NTf_2_]^−^ (100). IR ν_max_/cm^−1^: 3162(w), 2943(w), 2833(w), 1346(s), 1180(s), 1098(s), 1050(s). Elemental analysis (%); calculated for C_10_H_15_N_3_O_6_S_2_F_6_: C, 26.60; H, 3.35; N, 9.31. Found: C, 26.02; H, 3.30; N, 9.52.

1-Methoxymethoxyethyl-3-methyl imidazolium bis(trifluoromethanesulfonyl)imide (MME). The synthesis of MME was performed by modifying a previous procedure [32]. 1-Bromo-2-(methoxymethoxy)ethane (8.69 g, 51.40 mmol) was added dropwise to 1-methyl imidazole (4.20 g, 51.20 mmol) dissolved in dry acetone (40 mL). The reaction mixture was refluxed at 65 °C for 48 h before washing with acetone (≈50 mL) and removing of volatiles in vacuo. A solution of Li[NTf_2_] (14.53 g, 52.20 mmol) in distilled water (20 mL) was then added and the reaction mixture left stirring at room temperature for 24 h. The resulting IL (lower phase) was then separated and washed several times with distilled water (≈100 mL) until no precipitate of AgBr formed when aq. AgNO_3_ was added to the washing water. Remaining water was removed by stirring the IL under vacuum (≈0.05 mbar) at 60 °C overnight. Yield: 19.16 g (83%).

^1^H NMR (400 MHz, CDCl_3_): δ (ppm) 8.77 (s, 1H), 7.42 (t, 1H), 7.28 (t, 1H), 4.61 (s, 2H), 4.39 (t, 2H), 3.95 (s, 3H), 3.86 (t, 2H), 3.30 (s, 3H). ^13^C NMR {^1^H} (400 MHz, CDCl_3_): δ (ppm) 136.65, 123.17, 121.37, 118.17, 96.54, 65.20, 55.61, 50.12, 36.41. ^19^F NMR (400 MHz, CDCl_3_): δ (ppm) −81.00 (s, CF_3_). ESI-MS: positive, ([%]) *m*/*z* 171.1 [MMEIm]^+^ (100); negative, ([%]) *m*/*z* 279.9 [NTf_2_]^−^ (100). IR ν_max_/cm^−1^: 3162(w), 2943(w), 2833(w), 1346(s), 1180(s), 1098(s), 1050(s). Elemental analysis (%); calculated for C_10_H_15_N_3_O_6_S_2_F_6_: C, 26.61; H, 3.35; N, 9.31. Found: C, 26.10; H, 3.02; N, 9.52.

1-Butylcyano-3-methylimidazolium bis(trifluoromethanesulfonyl)imide (CN). The synthesis of CN was carried out following a previously described procedure [33]. 5-Chlorovaleronitrile (8.46 g, 71.90 mmol) was slowly added to 1-methyl imidazole (4.92 g, 59.90 mmol) and the mixture stirred at 80 °C for 4 h. Next, the temperature was increased to 110 °C for 24 h where after the resulting brownish-yellow liquid was washed several times with diethyl ether (≈50 mL) and then decolorized by stirring with a mixture of activated charcoal (0.7 g, 58.2 mmol) in distilled water (10 mL) overnight. After removal of the activated charcoal by filtration, Li[NTf_2_] (20.09 g, 69.9 mmol) in distilled water (20 mL) was added and the reaction mixture stirred at room temperature for 24 h. The resulting IL (lower phase) was then separated and washed several times with distilled water (≈100 mL) until no precipitate of AgCl formed when aq. AgNO_3_ was added to the washing water. Remaining water was removed by stirring the IL under vacuum (≈0.05 mbar) at 60 °C overnight. Yield: 21.27 g (80%).

^1^H NMR (400 MHz, DMSO-d_6_): δ (ppm) 8.97 (s, 1H), 7.94 (s, 1H), 7.90 (s, 1H), 4.72 (t, 2H), 4.38 (s, 3H), 2.50 (m, 2H), 2.19 (m, 2H). ^13^C NMR {^1^H} (400 MHz, DMSO-d_6_): δ (ppm) 135.47, 123.79, 122.26, 48.68, 35.88, 28.69, 21.79, 16.07. ^19^F NMR (400 MHz, DMSO-d_6_): δ (ppm) −80.40 (s, CF_3_). ESI-MS: positive, ([%]) *m*/*z* 164.1 [C_4_CNIm]^+^ (100); negative, ([%]) *m*/*z* 279.9 [NTf_2_]^−^ (100). IR ν_max_/cm^−1^: 3161(w), 2963(w), 2252(w), 1346(s), 1180(s), 1050(s). Elemental analysis (%); calculated for C_11_H_14_N_4_O_4_S_2_F_6_: C, 29.73; H, 3.10; N, 12.61. Found: C, 29.70; H, 2.75; N, 12.56. 

### 2.4. Synthesis of FIL-Stabilized Ru Nanoparticles (RuNPs/FIL)

In a typical synthesis, [Ru(COD)(COT)] (9.40 mg, 0.03 mmol) and a given IL (1 mL) were introduced in a Fischer-Porter reactor under argon atmosphere, and the mixture stirred (1500 rpm) at 40 °C for 24 h. The so-obtained homogeneous yellow mixture was then exposed to 3 bar H_2_ in dynamic flow for 10 min and then in a static mode for 22 h at room temperature, except for CN, for which a temperature of 60 °C was applied. Next, generated cyclooctane was removed under vacuum (≈0.05 mbar) and the resultant black colloidal suspension containing RuNPs/FIL stored under inert atmosphere inside a glovebox prior to analyses and/or catalytic reactions.

### 2.5. Catalytic Hydrogenation of Styrene

Catalytic hydrogenation of styrene (S) was performed in a 20 mL Fischer-Porter reactor under 5 bar of H_2_ pressure at 30 °C using a stirring rate of 1500 rpm. In a typical experiment, a mixture containing a given RuNPs/IL catalyst (0.02 mmol of metal), styrene (0.5 mL, 4 mmol) and octane (0.05 mL, 0.3 mmol) as internal standard was loaded in the reactor. Aliquots of the reaction mixture were taken at different intervals of time for quantitative gas chromatography (GC) analysis to follow the evolution of each catalytic test. 

Recyclability tests were performed after washing the spent RuNPs/IL catalyst three times with pentane (3 × 4 mL), to remove substrate and formed products, followed by drying under vacuum (≈0.05 mbar) at room temperature. The dried RuNPs/ILs phase was then mixed with a new batch of substrate and the reaction performed under the same catalytic conditions as mentioned above. This operation was repeated five times. TEM and ICP analyses were performed on the spent RuNPs/ILs. 

### 2.6. Product Analysis from Catalytic Reactions

Quantitative analysis on the mixtures from the catalytic reactions was performed by a GC with flame ionization detection (FID) using the internal standard method. Calibration curves were obtained with commercial reference products. GC analyses were performed on a Shimadzu GC-2010 equipped with a Zebrom-zb-5ms capillary column (30 m × 0.25 mm × 0.25 μm) using He as the carrier gas (He flow: 1.25 mL/min; injector temperature: 250 °C; detector (FID) temperature: 250 °C; oven program: 50 °C (hold 3 min) to 240 °C at 10 °C/min (hold 10 min) for a total run time of 30 min. Retention times: octane 2.6 min; ECH 3.1 min; S 9.4 min; EB 7.1 min) or on an Agilent 6850-5975C using an HP-5MS capillary column (30.0 m × 250 μm × 0.25 μm) with He carrier gas (He flow: 1.25 mL/min; injector temperature: 250 °C; detector (FID) temperature: 250 °C; oven program: 50 °C (hold 3 min) to 240 °C at 10 °C/min (hold 10 min) for a total run time of 30 min. Retention times: octane 4.1 min; ECH 4.6 min; EB 5.1 min; S 5.5 min).

### 2.7. Solubility Measurements

The solubility of styrene (S) and ethylbenzene (EB) in the different ILs was determined through the following method: A mixture of IL (0.5 mL) and S or EB (2 mL) was stirred at room temperature for 24 h in a vial and then allowed to settle for 0.5 h. The excess of S or EB present on the top of the mixture was removed via cannula filtration. Then, pentane (5 mL) was added and the mixture vigorously stirred to extract the dissolved S or EB from the IL to the pentane. The pentane phase was analyzed by GC using octane (0.03 mmol) as internal standard. This extraction procedure was repeated twice, and the quantitative GC data allowed to determine the concentration of S and EB dissolved in each IL (see Supporting Information Table S3).

## 3. Results and Discussion

### 3.1. Synthesis and Characterization of RuNPs/ILs

**Scheme 1:**
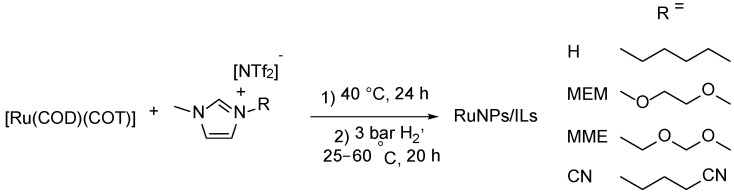
Synthesis procedure of RuNPs/ILs.

**Figure 1:**
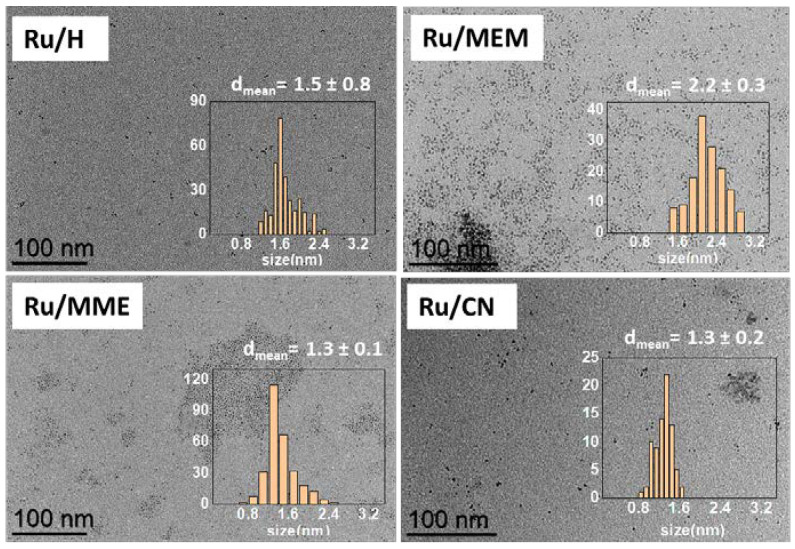
TEM images of Ru/H, Ru/MEM, Ru/MME and Ru/CN with their corresponding size distribution (scale bar = 100 nm).

Three FILs (MEM, MME, CN) and a corresponding unfunctionalized IL (H) (Table 1) were prepared in good to excellent yields by anion metathesis from their respective halide salts with Li[NTf_2_] following previously described, or slightly modified, procedures [30,31,32,33]. After purification and drying in vacuo, the ILs were thoroughly characterized using liquid NMR (^1^H, ^13^C{^1^H}, ^19^F, ^13^C dept-135), ATR-IR, ESI-MS, EA and TGA techniques (for detailed spectra and other characterization data see Supporting Information Figures S1–S17). For all ILs, the water content was below 50 ppm (detection limit of the Karl Fischer titration). RuNPs/IL systems were then synthesized by decomposing [Ru(COD)(COT)] in the ILs under 3 bar H_2_ and room temperature, except for the nitrile-FIL (CN) where complete decomposition of the Ru complex required a higher temperature of 60 °C (Scheme 1) [22]. This procedure led to the formation of well-dispersed RuNPs in all the ILs with a mean size ranging from 1.3 to 2.2 nm as observed by TEM analyses (Table 1 and Figure 1; HRTEM and HAADF-STEM images shown in Supporting Information Figures S18–S21. The metal content of each batch of RuNPs/IL was established by ICP analysis and found to be close to the expected value of 0.2 wt.% Ru (Table 1).

**Figure 2:**
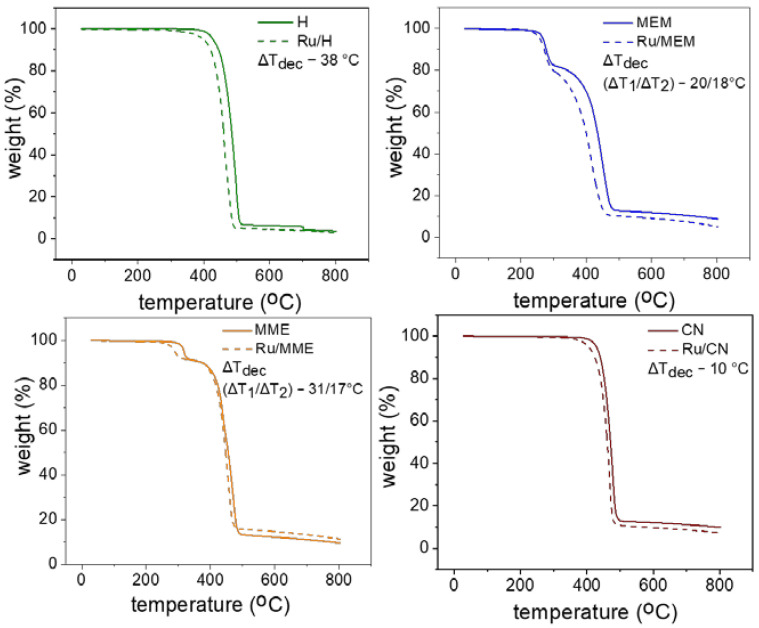
TGA profiles of the FILs (solid lines) and corresponding RuNPs/ILs (dotted lines).

It is known that the side chain of the cation and the nature of the anion play a crucial role in determining the thermal stability of IL [34]. Thus, TGA of all the ILs and the RuNPs/ILs were performed under N_2_ to compare their stability (Figure 2 and Supporting Information S22) [35]. The H and CN FILs proved to be most thermally stable, presenting the highest temperatures of decomposition (T_dec_) of 404 °C and 454 °C, respectively. In contrast, MEM and MME showed an initial weight loss, attributed to the ether functionalities, between 230–320 °C (18.6 wt.%) and 308–360 °C (7.8 wt.%), respectively, evidencing that the methoxy-containing FILs were thermally less stable. Notably, the RuNPs/ILs showed a lower T_dec_ for all ILs, thus indicating interaction between the ILs and the RuNPs. The difference in decomposition temperature (ΔT_dec_) of the ILs, in the presence or the absence of RuNPs, was the highest for H (Figure 2). This may result from steric stabilization of the RuNPs inside the non-polar domains of H [3], leading to a maximum proximity of the alkyl chains of H with the Ru surface and favoring their decomposition. Conversely, the smallest ΔT_dec_ was observed for CN in which the RuNPs are expected to interact strongly through the CN functionality, and therefore have less influence on the decomposition of the alkyl chain compared to the H counterpart [22,36,37,38]. Concerning the two methoxy-FILs, MEM and MME, the ΔT_dec_ were found between those of H and CN, thus indicating some interaction between the surface of the RuNPs and the ILs but less pronounced than with CN. Hence, overall, the RuNPs/ILs colloidal suspensions remained robust when exposed to high temperature.

XPS analyses were also performed on the ILs and RuNPs/ILs and the corresponding survey spectra along with deconvoluted high-resolution scan spectra of the different elements (C 1s, N 1s, F 1s, S 2p, O 1s, Ru 3p) are compiled in Supporting Information, Figures S23–S32. The elemental binding energies in the ILs (E_IL_) were consistent with literature data [39,40]. A noticeable shift in some binding energies was observed in the presence of RuNPs (E_RuIL_) as represented by ΔE and summarized in Table 2 for the different elements (all data shown in Supporting Information Tables S1 and S2). For H, the ΔE_C1s_ was more pronounced for C_2_ and C_3_ of the cation (Table 2, Supporting Information Figure S24), while it was lower for the elements present in the [NTf_2_]^−^ anion of the IL. This could indicate a preferred solvation of RuNPs in the non-polar domains of H with weak associated interactions. Conversely, a higher ∆E was found for N 1s, F 1s, O 1s, and to some extent S 2p, for the elements in the anions of MEM, MME and CN, which may be attributed to the presence of both anions and cations near the surface of the RuNPs [23]. The O 1s_cation_ and O 1s_anion_ in MEM and MME displayed similar binding energies, and hence, only a single peak was observed [41,42]. The higher values of ∆E_O1s_ for MEM and MME compared to H and CN could also be due to the stabilization of RuNPs by the oxygen atoms from the cation in addition to the ones from the anions. A change in the E_C1s_ of C_4_ to a lower value in the presence of RuNPs (i.e., negative ΔE) would indicate the formation of a nucleophilic species, e.g., *N*-heterocyclic carbene derived from the imidazolium cation [43]. However, for all ILs, ∆E_C1s_ of C_4_ was positive, suggesting that carbene formation during RuNPs synthesis was negligible. It is likely that the very low basicity of the anion [NTf_2_]^−^ and the mild RuNPs synthesis conditions reduced the reactivity between the moderately acidic C_4_ proton and the anion. Despite the very low concentration of RuNPs in the ILs, an increased number of scans enabled us to also acquire Ru 3p_3/2_ spectra (Supporting Information, Figure S32), where metallic Ru(0) (460.7 eV) and oxidized species Ru(IV)O_2_, hydrous Ru(IV)O_2_ and Ru(III)O_3_ (likely formed during sample handling in air) with distinct bonding energies were identified. Notably, for all the RuNPs/ILs, the bonding energy of the Ru(0) was lower than the reported values for metallic Ru (461.7 eV) in the literature [44]. These results further corroborate that the RuNPs interacted with the ILs. 

### 3.2. Catalysis with RuNPs/ILs

**Scheme 2:**
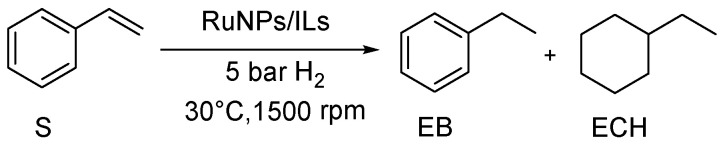
Hydrogenation of styrene (S) to ethyl benzene (EB) and ethyl cyclohexane (ECH).

The catalytic performances of the RuNPs/ILs systems were investigated for the hydrogenation of styrene (S) to form ethylbenzene (EB) and ethylcyclohexane (ECH) using mild reaction conditions (30 °C, 5 bar H_2_, S:Ru ratio 200:1) and vigorous stirring (1500 rpm) to avoid mass transfer issues during reaction with the moderately viscous IL phases (Scheme 2). Table 3 summarizes the catalytic results obtained. Note that the ILs alone did not show any conversion of S when submitted to the same reaction conditions.

**Figure 3:**
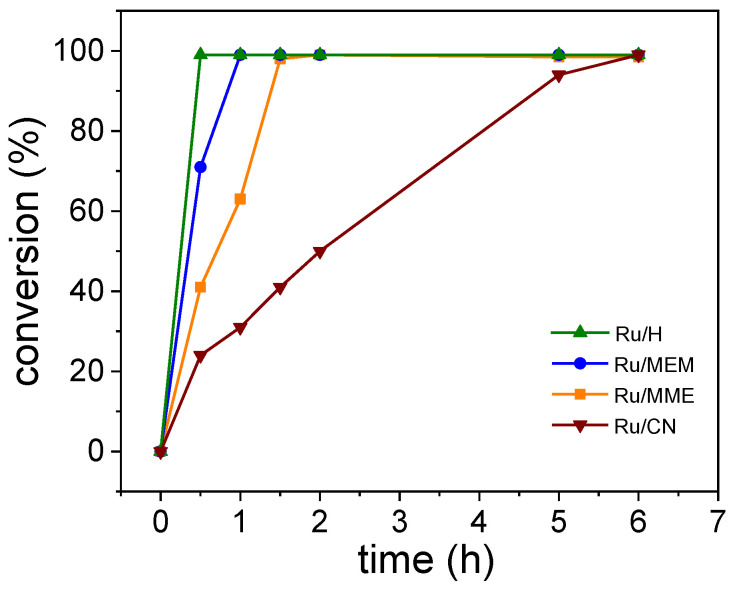
Time–conversion curves for the hydrogenation of styrene with the different RuNPs/ILs catalysts. Reaction conditions: 0.02 mmol of Ru, 4 mmol of styrene, 0.3 mmol of octane (internal standard), 5 bar H_2_, 30 °C, 1500 rpm.

As shown in Table 3, all the RuNPs/ILs systems were active for the catalytic hydrogenation of S, providing full substrate conversion in short reaction times (0.5–6 h) but with a difference in activity as a function of the IL. The difference in the conversion rate vs. the RuNPs/ILs systems (Figure 3 and Supporting Information Figures S34–S37) was more deeply analyzed through the calculation of the turnover frequencies (TOF) at isoconversion (see Supporting Information Table S4). The highest TOF value (1332 h^−1^) was reached with the RuNPs embedded in the non-functionalized IL (Ru/H catalyst). The two methoxy-functionalized IL systems (Ru/MEM and Ru/MME) yielded high conversions (TOFs of 629 and 212 h^−1^, respectively), while the Ru/CN had the lowest activity (TOF of 10 h^−1^). These differences in reaction rates may be correlated to the variation in polarity of the ILs that can influence the solubility of the substrate, with styrene expected to be more soluble in non-polar domains than in polar ones. Actually, the H IL containing only alkyl chains in its structure presented the larger non-polar domains of the IL series [37], and the RuNPs stabilized in this IL led to the highest TOF. Conversely, the more polar IL is expected to be the CN FIL, and accordingly, the CN-stabilized RuNPs displayed the lower activity. To validate the correlation between the conversion results and the solubility of styrene in the RuNPs/ILs, solubility tests of the substrate in the different ILs were performed. The determined values are reported in Supporting Information, Table S3 and plotted against the TOF values in Supporting Information, Figure S33. The solubility of styrene in each IL appeared to correlate well with the conversion results and the calculated TOFs; the more efficient catalytic systems were those that allowed the higher solubility of the styrene, with the activity order Ru/H > Ru/MEM ≈ Ru/MME > Ru/CN.

In addition to displaying efficient catalytic activity in the hydrogenation of S, all RuNPs/ILs achieved total selectivity towards EB at full conversion. It is noteworthy that even after 24 h, Ru/MME and Ru/CN catalysts maintained total selectivity towards EB, while some successive hydrogenation of EB to ECH occurred for both Ru/H (7%) and Ru/MEM (22%). Moreover, for the latter system, the hydrogenation seemed to be faster as 4% ECH was obtained after 2 h (Supporting Information Figure S35). These results proved that the RuNPs/ILs catalysts were highly selective for the hydrogenation of the vinyl group of S, and were generally reluctant to hydrogenate the aromatic ring under the applied reaction conditions. This is markedly different to the previous literature where RuNPs were reported to hydrogenate aromatics even at room temperature [45,46,47]. Given the results obtained with the RuNPs/ILs catalysts, the solubility of EB in the ILs was measured to evaluate the influence of this parameter on the conversion of EB into ECH. As shown in Supporting Information Table S3, the solubility of EB was found to be similar to S in the ILs, thus corroborating that the high selectivity observed towards EB was not caused by the low solubility of EB in the ILs. Other key parameters can be the size of the RuNPs and/or the accessibility of the EB at the Ru surface that may be limited due to interaction between the ILs and the surface of the RuNPs. The hydrogenation of aromatics with Ru requires the presence of facets with an estimated minimum number of close and free Ru atoms of approximately three, which is favored in large and better crystallized NPs [48,49]. As the Ru/H and Ru/MEM catalysts contained the larger RuNPs (mean size of 1.5 and 2.2 nm, respectively, see Table 1), this could account for the observed hydrogenation of EB into ECH with these catalysts in opposition to the others that displayed smaller RuNPs. Apart from an NP size effect, a limitation of the surface accessibility could derive from steric hindrance and/or blockage of Ru surface atoms, and also the ILs could have an electronic influence on the Ru surface. Unfortunately, NMR investigations on the RuNPs/ILs systems did not provide clear data to allow for a conclusion on such effects due to the presence of intense IL signals that overlapped other signals if any were present. The Ru/MME and Ru/CN catalytic systems were found to be less prone to hydrogenate EB into ECH, indicating that less of the Ru surface was accessible in these systems than in Ru/H and Ru/MEM. This is supported by the lower size of the RuNPs in these catalysts, but it could also result from the interaction of the MME and CN FILs via the -O-CH_2_-O- and -CN groups, respectively. Such interactions may, for steric and/or electronic reasons, influence the surface properties of the Ru/IL NPs in a way that limits the hydrogenation of the aromatic ring. 

**Figure 4:**
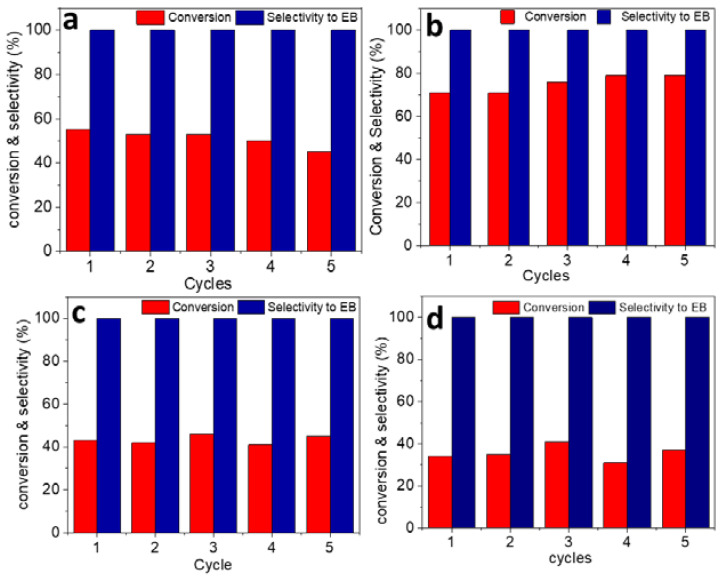
Recyclability tests of the RuNPs/ILs catalysts in the hydrogenation of styrene performed with different reaction times. (**a**) H (15 min), (**b**) MEM (30 min), (**c**) MME (30 min) and (**d**) CN (60 min). Reaction conditions: 0.02 mmol of Ru, 4 mmol of styrene, 0.3 mmol of octane (internal standard), 5 bar H_2_, 30 °C, 1500 rpm.

Recyclability of all RuNPs/ILs catalysts was tested in five successive catalytic runs (Figure 4). The recyclability tests were performed at partial conversions to easily identify the leaching of a catalyst, if any, and to provide a realistic evaluation of the catalytic performance. Notably, the styrene conversion with the RuNPs/H system decreased gradually during the five catalytic runs, whereas the three RuNPs/FILs systems maintained similar styrene conversions upon reuse. ICP analysis of the Ru/H phase from the first to the fifth run showed a decrease in the Ru content from 0.16 to 0.06 wt.%, thus confirming that Ru leaching from the IL phase during catalyst recycling was responsible for the lower conversion. In contrast, the Ru content remained quite unchanged during the five catalytic runs with the Ru/MEM, Ru/MME and Ru/CN systems. This could indicate that Ru-FIL interaction improved the confinement of the NPs in the RuNPs/FIL systems compared to Ru/H, and that the functionalization of IL had a positive influence on the stability of the catalysts [10]. Interestingly, TEM images of all spent Ru/IL catalysts (Supporting Information, Figure S38) showed no noticeable change in the mean size nor size distribution of the RuNPs compared to the pristine systems (Figure 1), pointing out that the RuNPs were stable under the reaction conditions applied in all cases and that leaching was not related to RuNPs’ instability. Hence, it is likely that the larger non-polar domains in the unfunctionalized IL compared to the FILs enhanced the catalyst solubility in the non-polar washing solvent (pentane), thus leading to a gradual catalyst loss during the washings between the catalytic runs. 

## 4. Conclusions

A series of catalysts based on RuNPs in non-functionalized IL (H) and FILs containing cyano (CN) and methoxy (MEM and MME) groups were synthesized, fully characterized and applied for the hydrogenation of styrene to study the influence of the nature of the IL on the catalytic properties. The RuNPs/FILs were found to be efficient and recyclable catalysts for the selective hydrogenation of styrene under milder conditions than previously reported in the literature. A difference in activity was achieved as a function of the nature of the IL, following the order Ru/H > Ru/MEM > Ru/MME >> Ru/CN. Interestingly, a total selectivity for ethyl benzene was also observed at a longer reaction time for the Ru/MME and Ru/CN systems, while the formation of ethyl cyclohexane occurred for Ru/H and Ru/MEM. This may result from a less accessible Ru surface for the phenyl group to coordinate in Ru/MME and Ru/CN, given the smaller RuNP sizes in these systems, or from metal-IL interaction of the MME and CN FILs via the -O-CH_2_-O- and -CN groups, respectively, as supported by the XPS data. Remarkably, the strong preference of RuNPs/ILs towards hydrogenation of the vinyl group vs. that of the aromatic ring highlights a reactivity pattern different than that for previously reported RuNPs. Overall, our study showed that the functionalization of ILs had a positive influence on the durability of the catalysts providing a good balance between stability and catalytic activity. Future work will focus on using the RuNPs/FILs for hydrogenation of alternative arene substrates such as, e.g., disubstituted CYE and ortho-para substituted styrene, which have previously been tested in the literature and thus would allow for a direct comparison of catalytic performance.

## Figures and Tables

**Table 1:** Metal content and mean size of the synthesized RuNPs.

Ionic Liquid	Ru Content (wt.%) ^a^	RuNPs/ILs	RuNP Mean Size (nm) ^b^
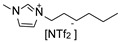	0.16	Ru/H	1.5 ± 0.8
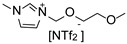	0.20	Ru/MEM	2.2 ± 0.3
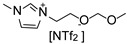	0.17	Ru/MME	1.3 ± 0.1
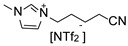	0.20	Ru/CN	1.3 ± 0.2

^a^ Determined by ICP analyses. ^b^ Determined from analyses of TEM images.

**Table 2:** Difference in binding energies (eV) of the chemical states of the elements in the ILs and RuNPs/ILs.

Ionic Liquid	ΔE Cation (eV)				ΔE Anion (eV)					
	C_2_ 1s	C_3_ 1s	C_4_ 1s	N 1s	C_5_ 1s	N 1s	F 1s	O 1s	S 2p_3/2_	S 2p_1/2_
H	0.10	0.27	0.07	0.09	0.07	0.16	0.12	0.04	0.15	0.17
MEM	−0.05	0.09	0.20	0.28	−0.08	0.23	0.29	0.41 *	0.11	0.14
MME	0.06	0.29	0.01	0.29	0.16	0.31	0.36	0.20 *	0.40	0.47
CN	0.01	-0.09	0.03	0.27	0.07	0.24	0.13	−0.04	0.35	0.28

* ΔE cation and anion.

**Table 3:** Hydrogenation of styrene with RuNPs/ILs catalysts ^a^.

RuNPs/ILs	Time (h)	Conversion (%) ^b^	Selectivity (%) ^b^		TOF (h^−1^) ^c^
			EB	ECH	
Ru/H	0.5	>99	>99	0	1332
Ru/H	24	>99	93	7	-
Ru/MEM	1	>99	>99	0	629
Ru/MEM	24	>99	78	22	-
Ru/MME	1.5	98	>99	0	212
Ru/MME	24	98	>99	0	-
Ru/CN	6	>99	>99	0	10
Ru/CN	24	>99	>99	0	-

^a^ Reaction conditions: 0.02 mmol of Ru, 4 mmol of styrene, 0.3 mmol of octane (internal standard), 5 bar H_2_, 30 °C, 1500 rpm. ^b^ Determined by GC using internal standard technique. ^c^ TOFs calculated at isoconversion (55%) with respect to the surface amount of metal (see Supporting Information Table S3 for details).

## Data Availability

Data are available from open access repositories, namely HAL for CNRS-LCC and Orbit for DTU.

## Supplementary Materials

The following supporting information can be downloaded at: https://www.mdpi.com/article/10.3390/nano13091459/s1, Figure S1: ^1^H NMR of H in CDCl_3_; Figure S2: ^13^C{^1^H} (bottom) and ^13^C{^1^H}-dept-135 (Top) NMR of H in CDCl_3_; Figure S3: ^19^F NMR of H in CDCl_3_; Figure S4: ^1^H NMR of MEM in CDCl_3_; Figure S5: ^13^C{^1^H} (bottom) and ^13^C{^1^H}-dept-135 (Top) NMR of MEM in CDCl_3_; Figure S6: ^19^F NMR of MEM in CDCl_3_; Figure S7: ^1^H NMR of MME in CDCl_3_; Figure S8: ^13^C{^1^H} (bottom) and ^13^C{^1^H}-dept-135 (Top) NMR of MME in CDCl_3_; Figure S9: ^19^F NMR of MME in CDCl_3_; Figure S10: ^1^H NMR of CN in DMSO-d6; Figure S11: ^13^C{^1^H} (bottom) and ^13^C{^1^H}-dept-135 (Top) NMR of CN in DMSO-d6; Figure S12: ^19^F NMR of CN in DMSO-d6; Figure S13: ATR-IR spectra of (a) H, (b) MEM, c) MME and (d) CN; Figure S14: (a) TGA and (b) DSC analyses of H; Figure S15: (a) TGA and (b) DSC analyses of MEM; Figure S16: (a) TGA and (b) DSC analyses of MME; Figure S17: (a) TGA and (b) DSC analyses of CN; Figure S18: (a) TEM image, (b) size histogram, (c) HRTEM image and (d) HAADF-STEM of Ru/H; Figure S19: (a) TEM image, (b) size histogram, (c) HRTEM image and (d) HAADF-STEM of Ru/MEM; Figure S20: (a) TEM image, (b) size histogram, (c) HRTEM image and (d) HAADF-STEM of Ru/MME; Figure S21: (a) TEM image, (b) size histogram, (c) HRTEM image and (d) HAADF-STEM of Ru/CN; Figure S22: TGA analyses of ILs (red lines) and of RuNPs/ILs (blue lines); Figure S23: Chemical structures of the ILs with numbering of atoms; Figure S24: (a) XPS survey spectrum of H and high-resolution scan spectra of (b) C 1s, (c) S 2p (d) F 1s, (e) N 1s and (f) O 1s; Figure S25: (a) XPS survey spectrum of MEM and high-resolution scan spectra of (b) C 1s, (c) S 2p (d) F 1s, (e) N 1s and (f) O 1s; Figure S26: (a) XPS survey spectrum of MME and high-resolution scan spectra of (b) C 1s, (c) S 2p (d) F 1s, (e) N 1s and (f) O 1s; Figure S27: (a) XPS survey spectrum of CN and high-resolution scan spectra of (b) C 1s, (c) S 2p (d) F 1s, (e) N 1s and (f) O 1s; Figure S28: (a) XPS survey spectrum of RuNPs/H and high-resolution scan spectra of (b) C 1s, (c) S 2p (d) F 1s, (e) N 1s and (f) O 1s; Figure S29: (a) XPS survey spectrum of RuNPs/MEM and high-resolution scan spectra of (b) C 1s, (c) S 2p (d) F 1s, (e) N 1s and (f) O 1s; Figure S30: (a) XPS survey spectrum of RuNPs/MME and high-resolution scan spectra of (b) C 1s, (c) S 2p (d) F 1s, (e) N 1s and (f) O 1s; Figure S31: (a) XPS survey spectrum of RuNPs/CN; and high-resolution scan spectra of (b) C 1s, (c) S 2p (d) F 1s, (e) N 1s and (f) O 1s; Figure S32: High-resolution XPS scan spectra of Ru 3p (a) H, (b) MEM, (c) MME and (d) CN; Figure S33: Solubility of S in the ILs (mmol/mL) vs. TOF/(surface atom); Figure S34: (a) S conversion and (b) selectivity into EB and ECH with time; Figure S35: (a) S conversion and (b) selectivity into EB and ECH with time; Figure S36: (a) S conversion and (b) selectivity into EB and ECH with time; Figure S37: (a) S conversion and (b) selectivity into EB and ECH with time; Figure S38: TEM images of the RuNPs/ILs systems (a) Ru/H, (b) Ru/MEM, (c) Ru/MME and (d) Ru/CN after five catalytic runs with their corresponding size distribution; Table S1: Binding energies (eV) of C 1s for the carbon atoms in the ILs and RuNPs/ILs; Table S2: Binding energies (eV) of the chemical states of the atoms in the ILs and RuNPs/ILs; Table S3: Solubility of S and EB in ILs; Table S4: Calculation of TOFs.
