# Supplementary material for: Ruthenium Nanoparticles Stabilized with Methoxy-Functionalized Ionic Liquids: Synthesis and Structure–Performance Relations in Styrene Hydrogenation
Source: Nanomaterials (Basel). 2023 Apr 25;13(9):1459. doi: 10.3390/nano13091459 (PMC10180216; doi:10.3390/nano13091459)
Supplement: Supplementary file 1 [file nanomaterials-13-01459-s001.zip › nanomaterials-2333940-supplementary.pdf]

Supporting information

Ruthenium Nanoparticles Stabilized with Methoxy-Functionalized Ionic Liquids: Synthesis and Structure–Performance Relations in Styrene Hydrogenation

Deepthy Krishnan [1,2,3], Leonhard Schill [1], M. Rosa Axet [2,3], Karine Philippot [2,3,*] and Anders Riisager [1,*]

[1] Centre for Catalysis and Sustainable Chemistry, Department of Chemistry, Technical University of
Denmark, DK-2800 Kgs. Lyngby, Denmark; deekr@kemi.dtu.dk (D.K.); leos@kemi.dtu.dk (L.S.)
[2] CNRS, LCC (Laboratoire de Chimie de Coordination), 205 Route de Narbonne, BP44099, CEDEX 4, 31077 Toulouse, France; rosa.axet@lcc-toulouse.fr
[3] Université de Toulouse, UPS, INPT, CEDEX 4, 31077 Toulouse, France
* Correspondence: karine.philippot@lcc-toulouse.fr (K.P.); ar@kemi.dtu.dk (A.R.)

Table of contents

1) NMR analysis of ionic liquids (ILs)

2) ATR-IR analysis of ionic liquids (ILs)

3) TGA and DSC analyses of Ionic Liquids (ILs)

4) TEM analysis of ruthenium nanoparticles stabilized in ILs (RuNPs/ILs)

5) TGA analysis of ILs and RuNPs/ILs

6) XPS analysis of ILs and RuNPs/ILs

7) Solubility test of styrene (S) and ethylbenzene (EB)

8) Hydrogenation catalysis with RuNPs/ILs

9) Calculation of TOF

10) TEM analyses of RuNPs/ILs after five catalytic runs

1. NMR analysis of ILs

1-Hexyl-3-methyl imidazolium bis(trifluoromethanesulfonyl)imide (H)

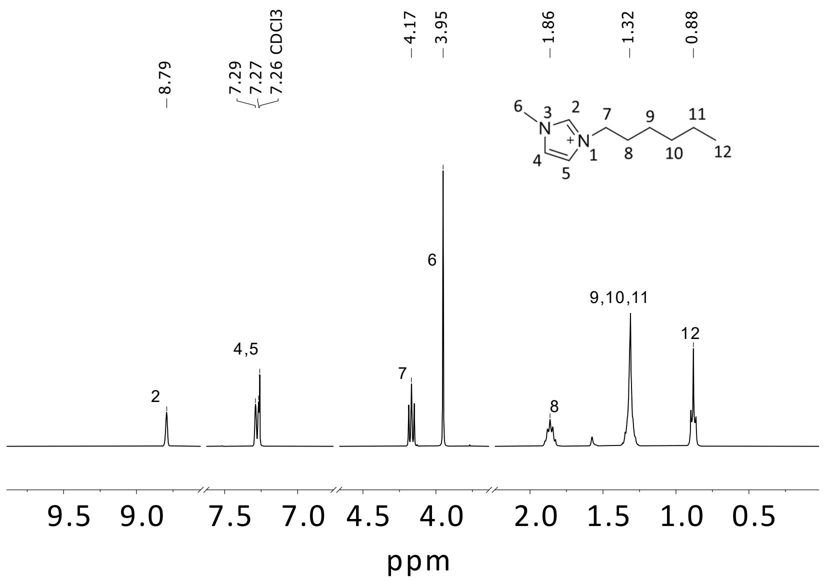

Figure S1. ^{1}H NMR of H in $CDCl_3$.

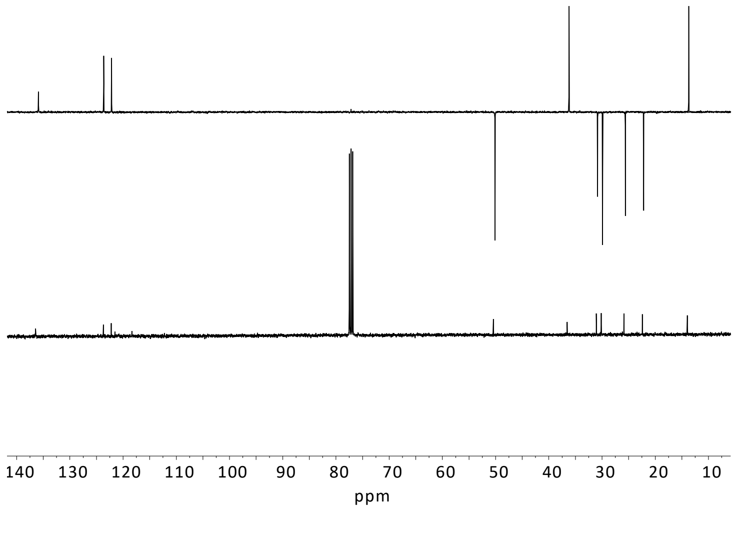

Figure S2. $^{13}C\{^{1}H\}$ (bottom) and $^{13}C\{^{1}H\}$-dept-135 (Top) NMR of H in $CDCl_3$.

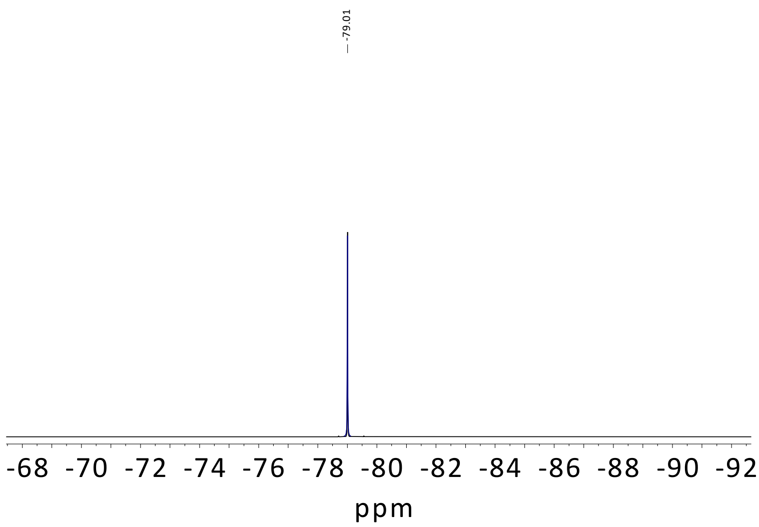

Figure S3. ^{19}F NMR of H in $CDCl_3$.

1-Methoxyethoxymethyl-3-methyl imidazolium bis(trifluoromethanesulfonyl)imide (MEM)

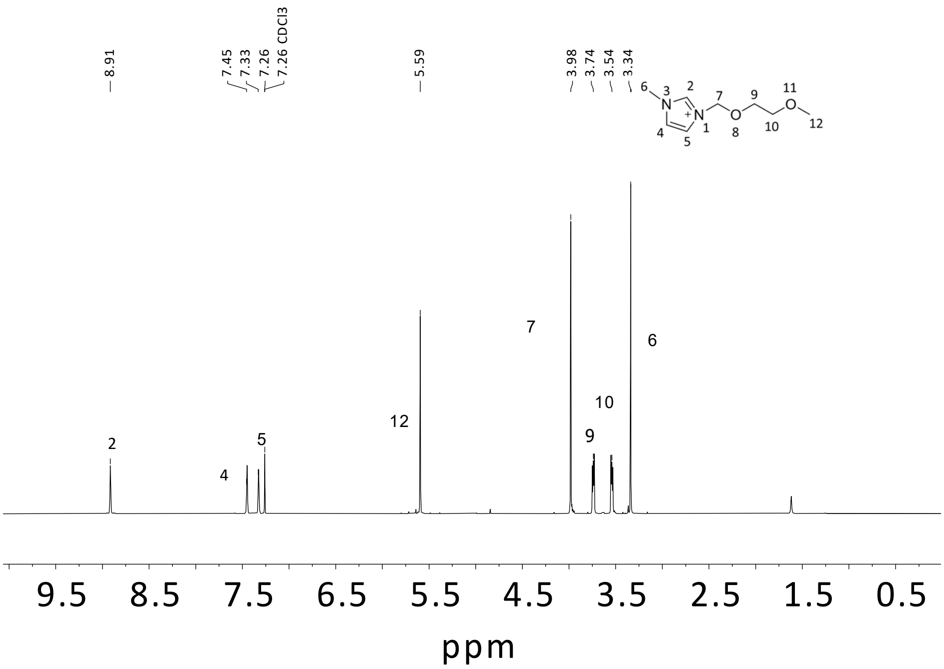

Figure S4. ^{1}H NMR of MEM in $CDCl_3$.

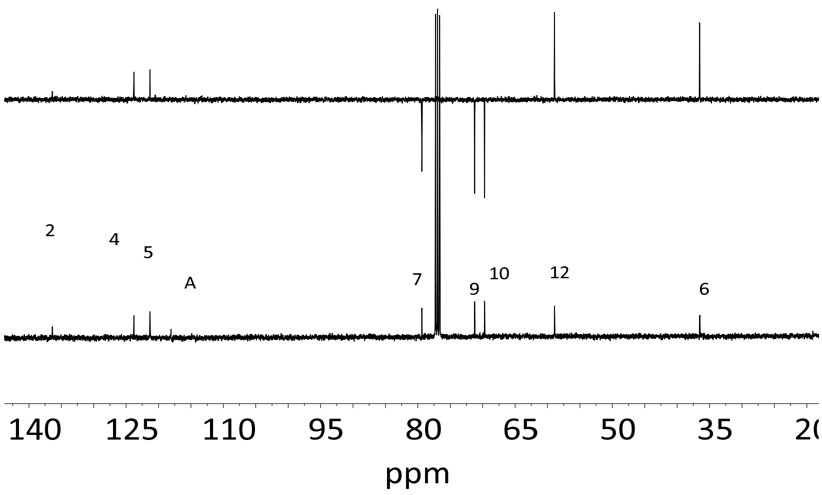

Figure S5. $^{13}C\{^1H\}$ (bottom) and $^{13}C\{^1H\}$-dept-135 (Top) NMR of MEM in $CDCl_3$.

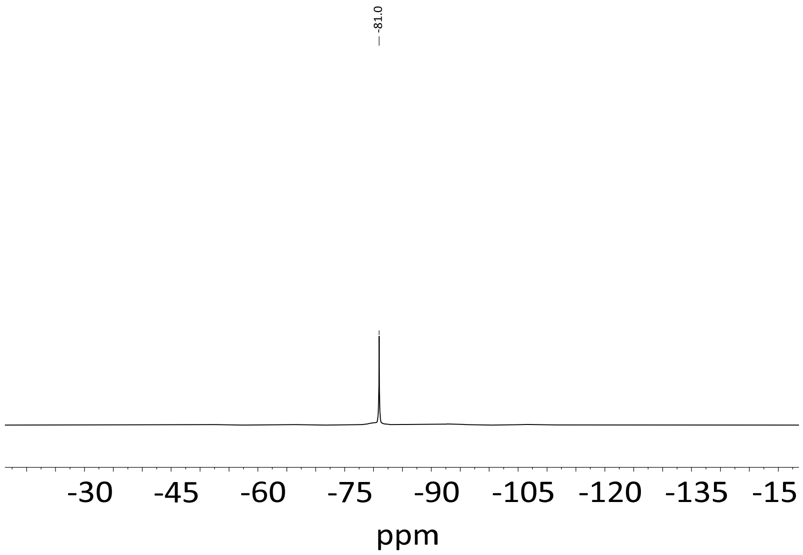

Figure S6. ^{19}F NMR of MEM in $CDCl_3$.

1-Methoxymethylethoxy-3-methyl imidazolium bis(trifluoromethanesulfonyl)imide (MME)

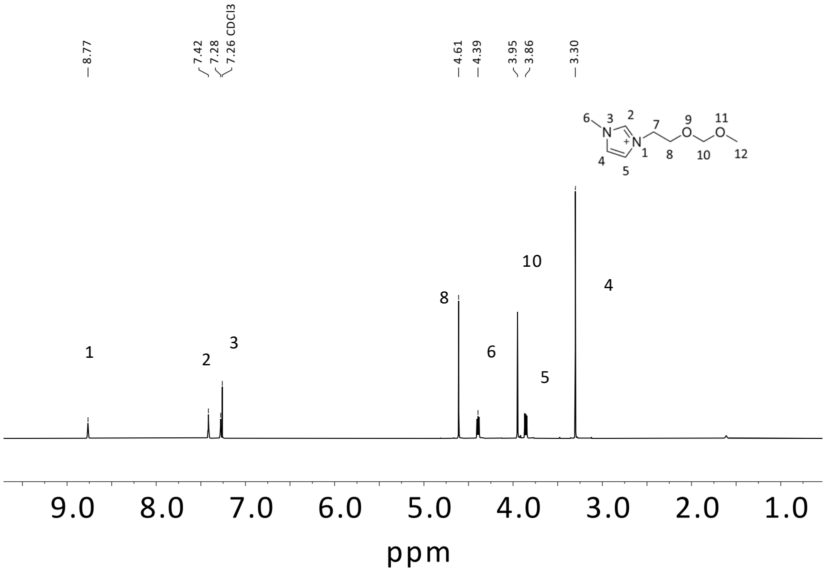

Figure S7. ^{1}H NMR of MME in $CDCl_3$.

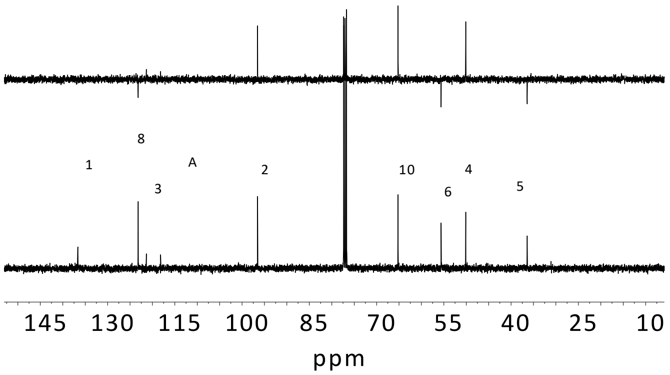

Figure S8. $^{13}C\{^1H\}$ (bottom) and $^{13}C\{^1H\}$-dept-135 (Top) NMR of MME in $CDCl_3$.

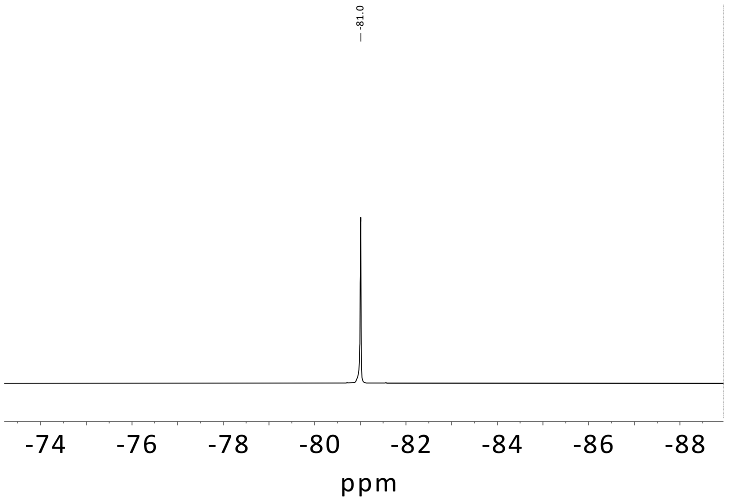

Figure S9. ^{19}F NMR of MME in $CDCl_3$.

1-Butylnitrile-3-methyl imidazolium bis(trifluoromethanesulfonyl)imide (CN)

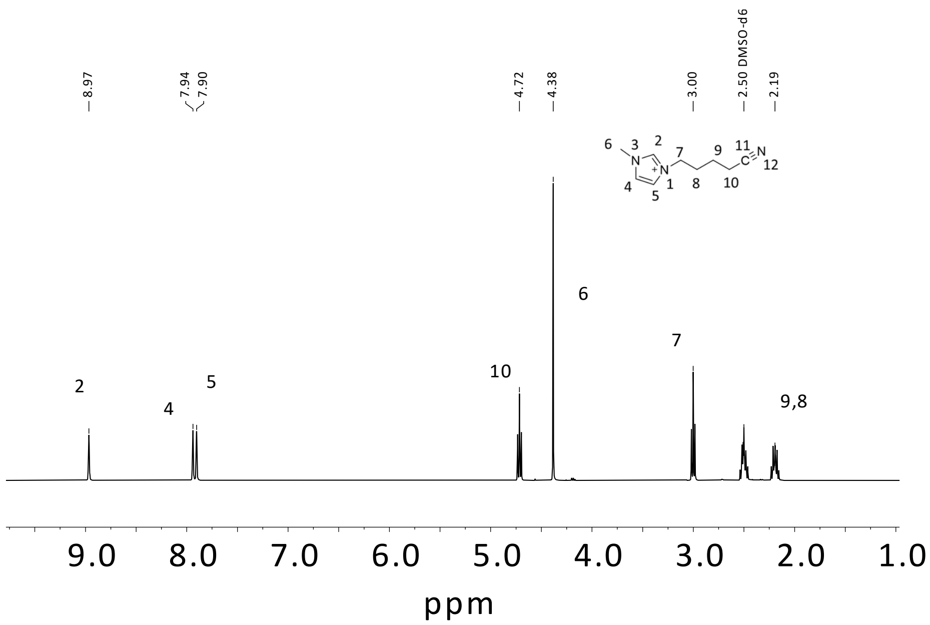

Figure S10. ^{1}H NMR of CN in DMSO-d_6.

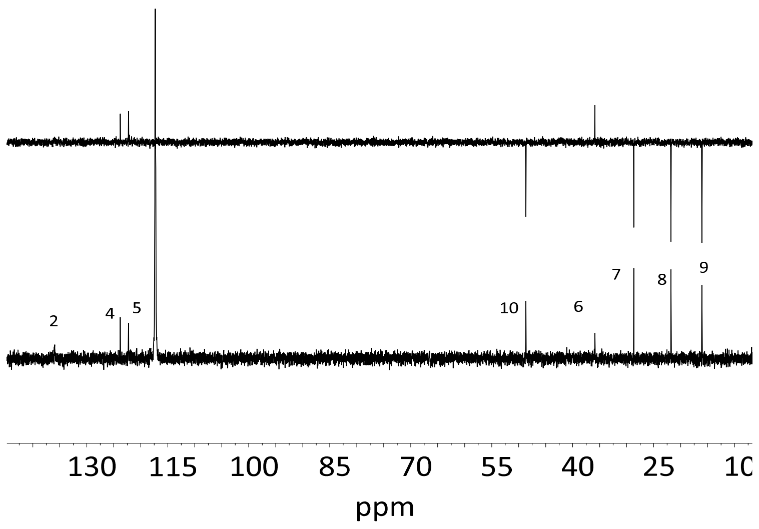

Figure S11. $^{13}C\{^1H\}$ (bottom) and $^{13}C\{^1H\}$-dept-135 (Top) NMR of CN in DMSO-d_6.

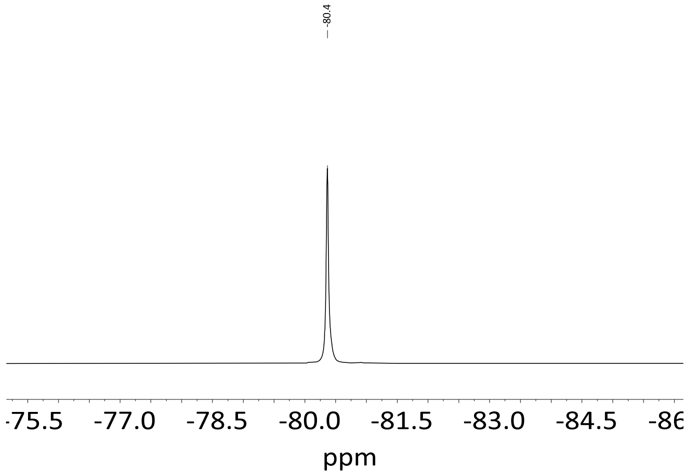

Figure S12. ^{19}F NMR of CN in DMSO-d_6.

2. ATR-IR analysis of ILs

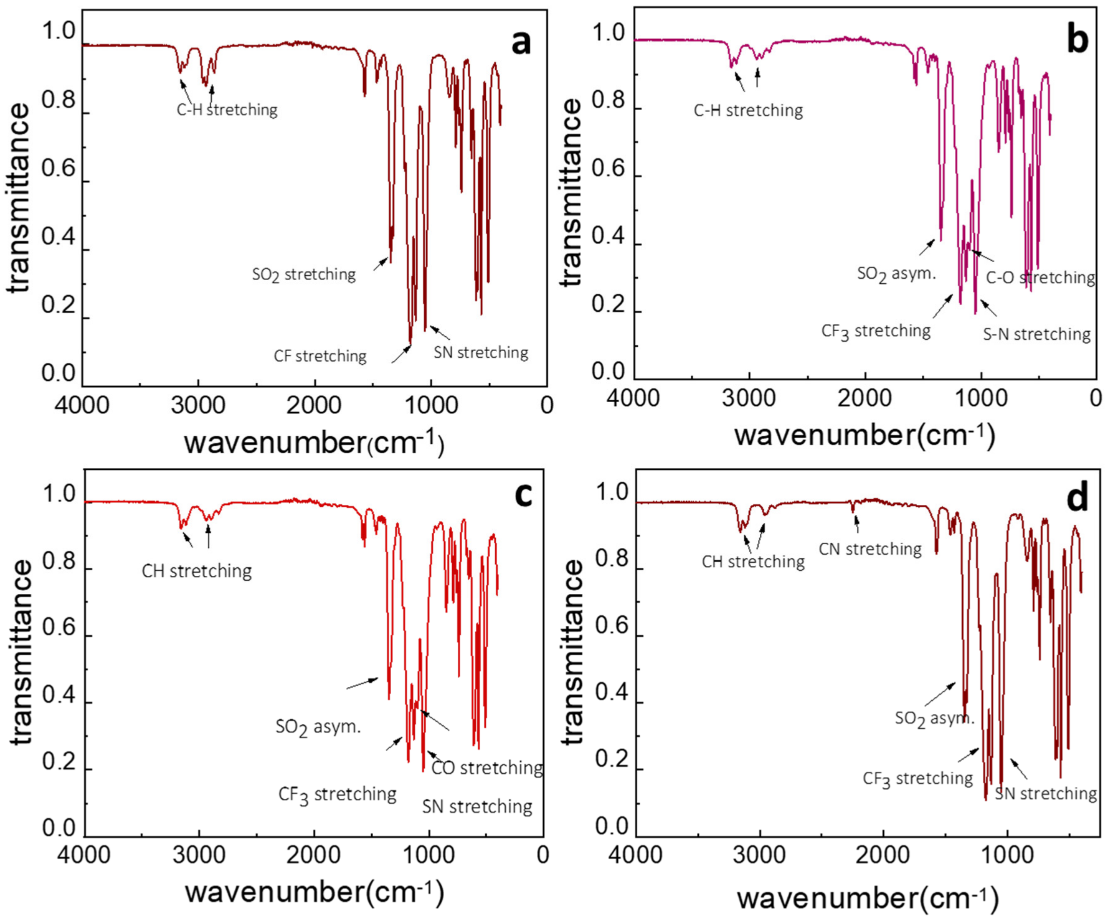

Figure S13. ATR-IR spectra of a) H, b) MEM, c) MME, and d) CN.

3. TGA and DSC analyses of ILs

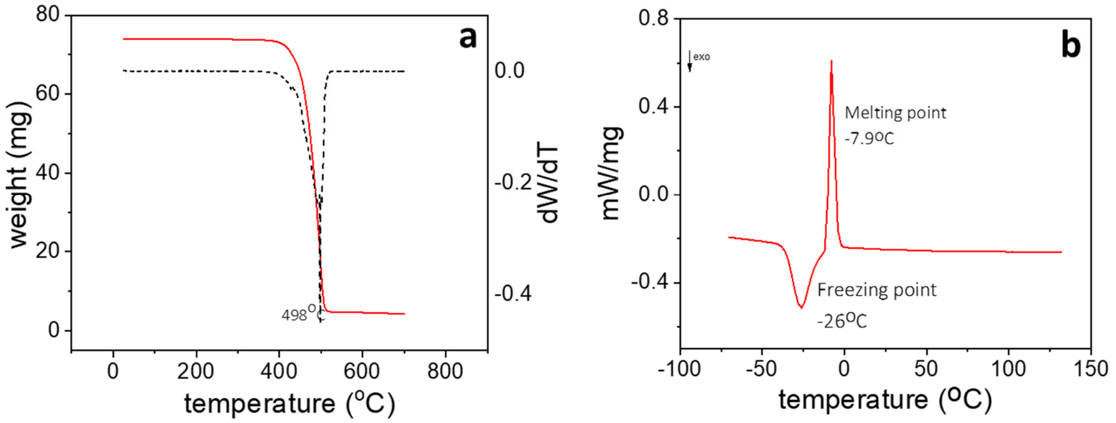

Figure S14. a) TGA and b) DSC analyses of H.

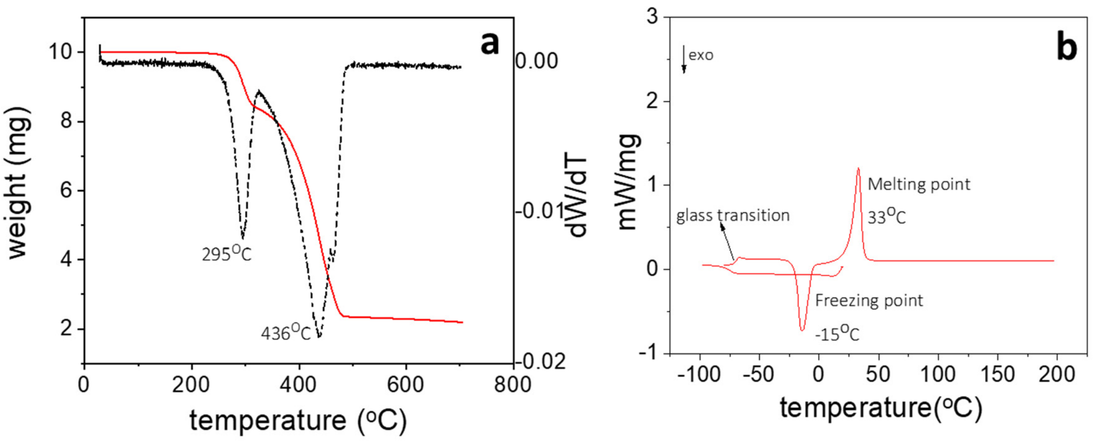

Figure S15. a) TGA and b) DSC analyses of MEM.

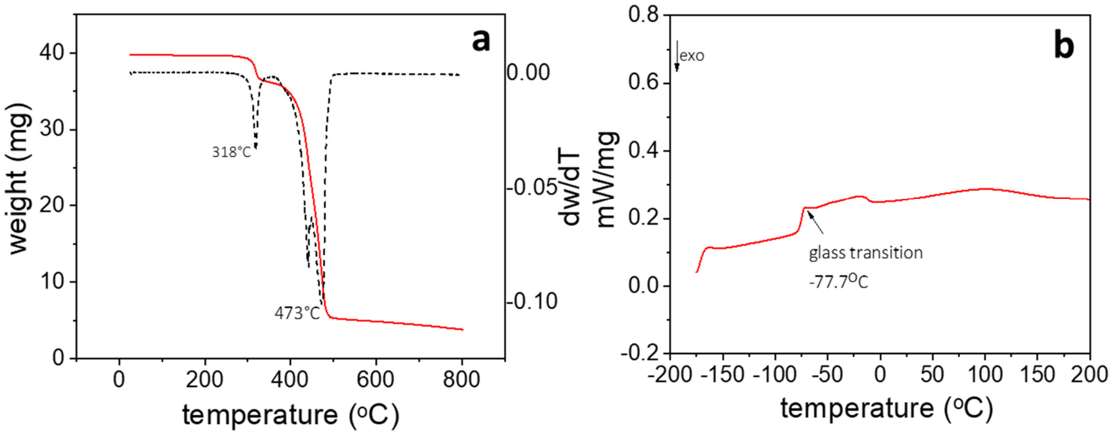

Figure S16. a) TGA and b) DSC analyses of MME.

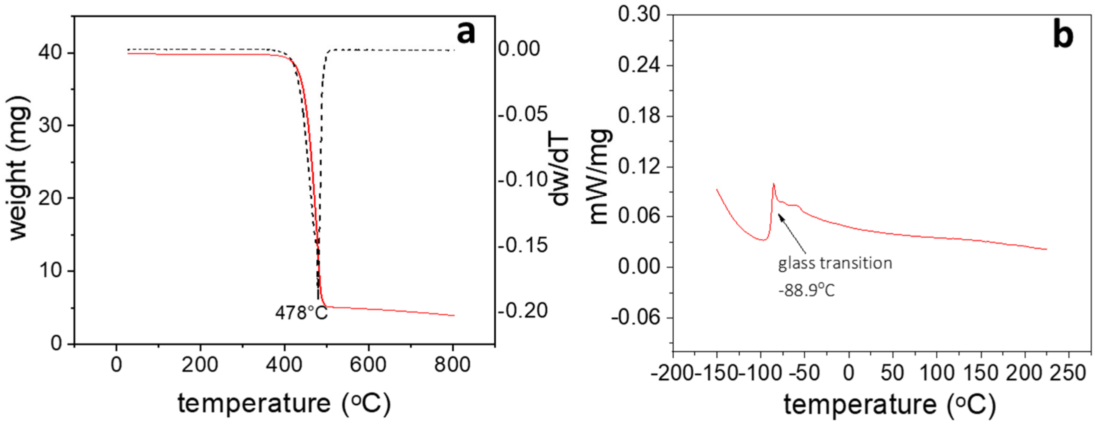

Figure S17. a) TGA and b) DSC analyses of CN.

4. TEM analysis of Ru/ILs

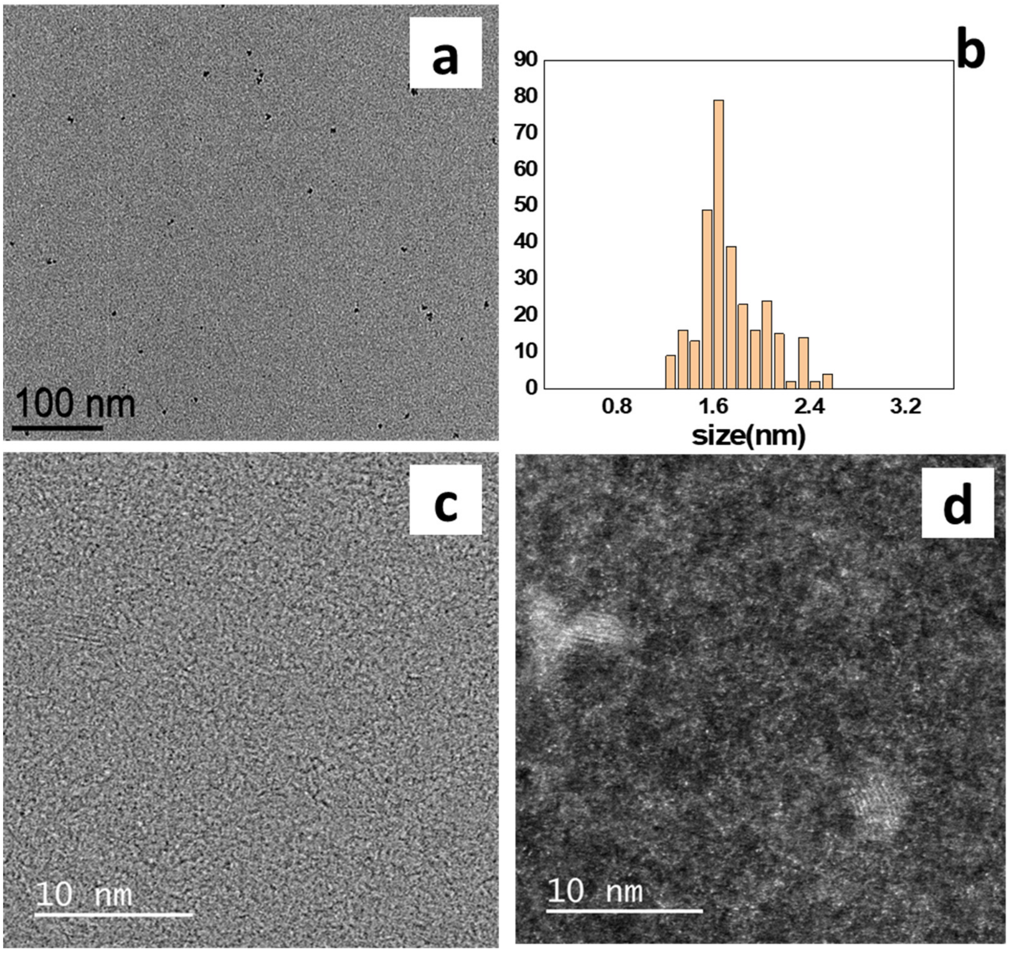

Figure S18. a) TEM image, b) size histogram, c) HRTEM image and d) HAADF-STEM of Ru/H.

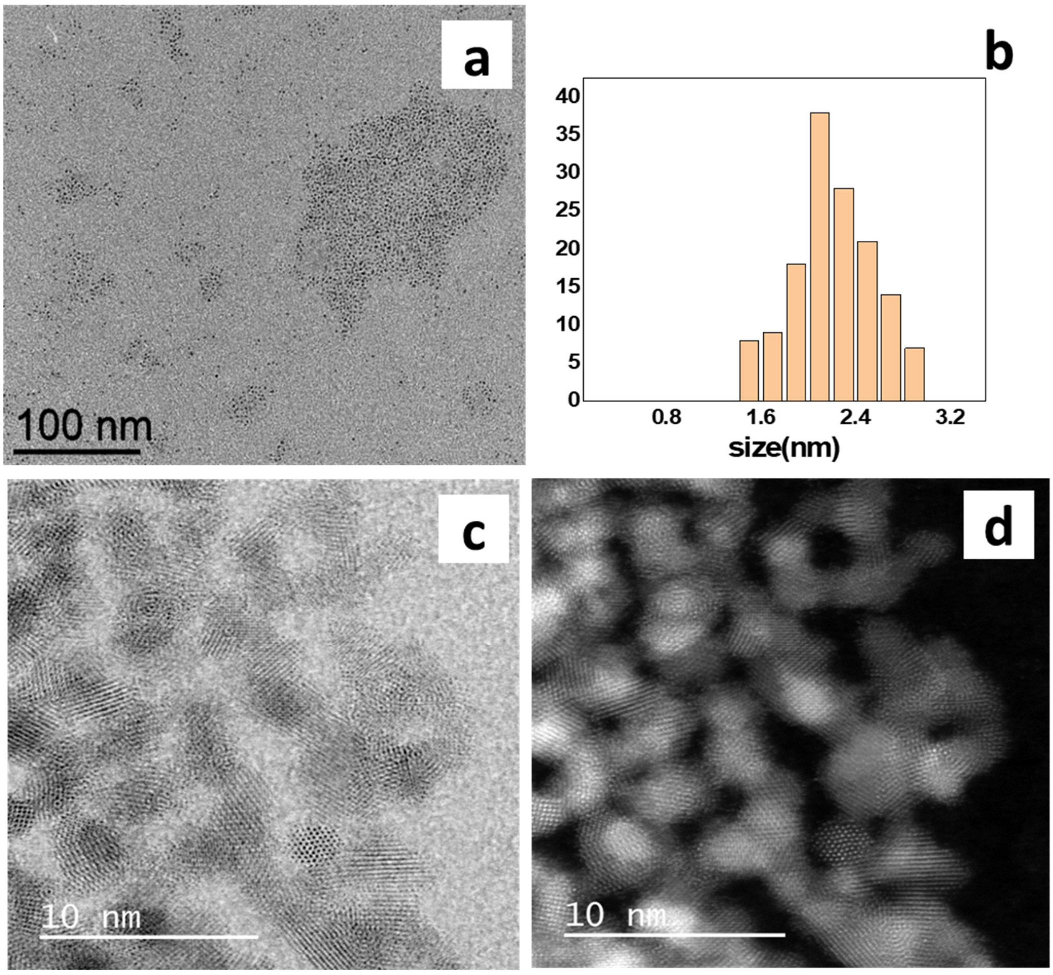

Figure S19. a) TEM image, b) size histogram, c) HRTEM image and d) HAADF-STEM of Ru/MEM.

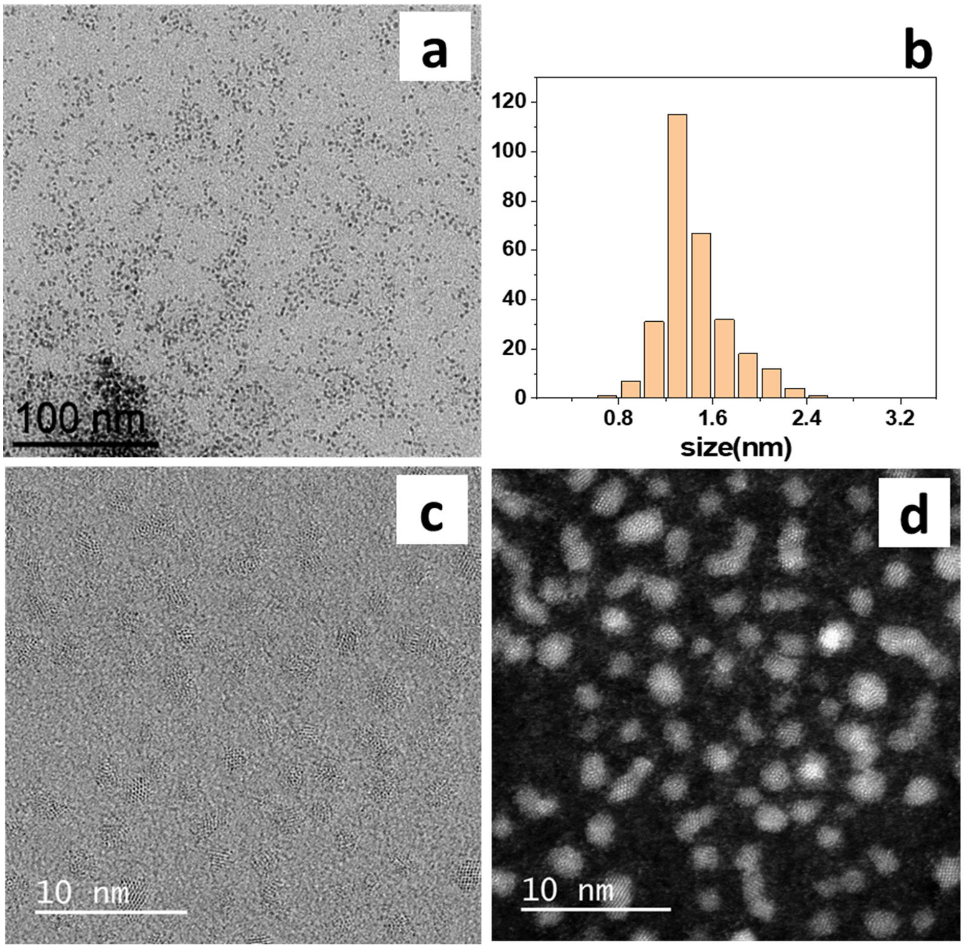

Figure S20. a) TEM image, b) size histogram, c) HRTEM image and d) HAADF-STEM of Ru/MME.

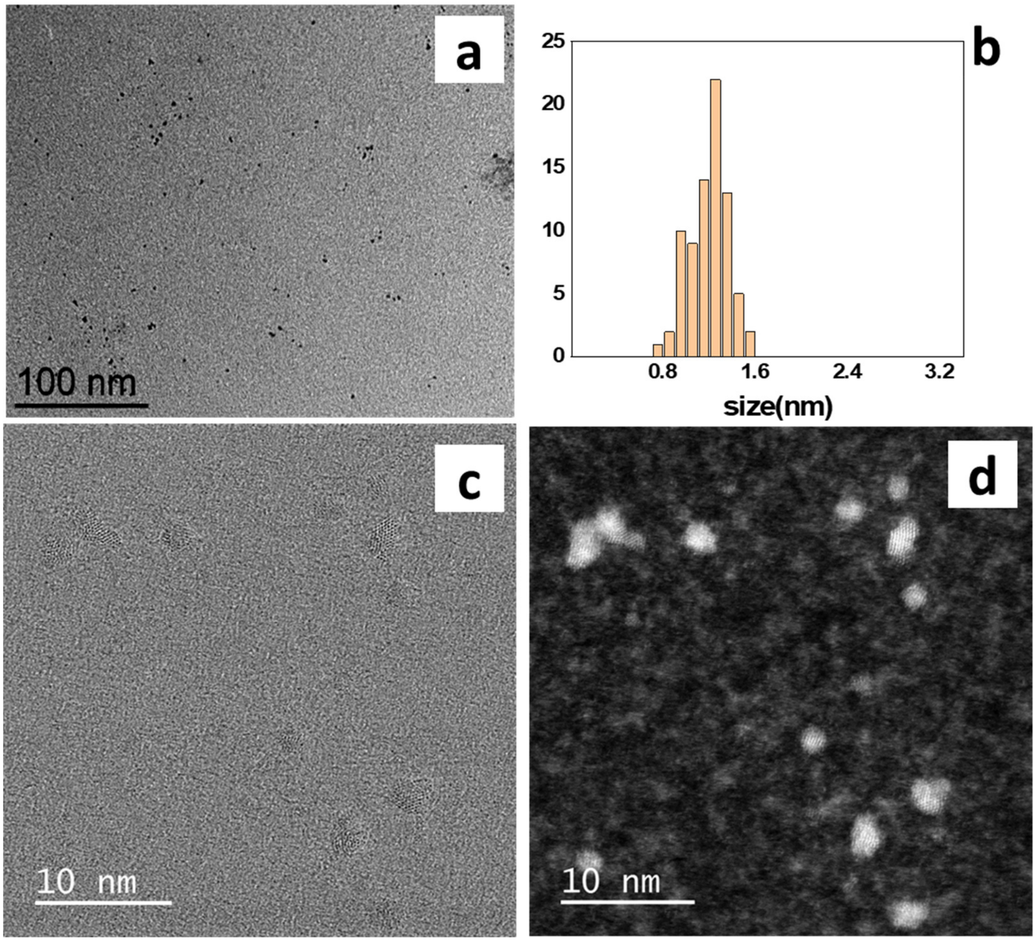

Figure S21. a) TEM image, b) size histogram, c) HRTEM image and d) HAADF-STEM of Ru/CN.

5. TGA analysis of IL and Ru/ILs

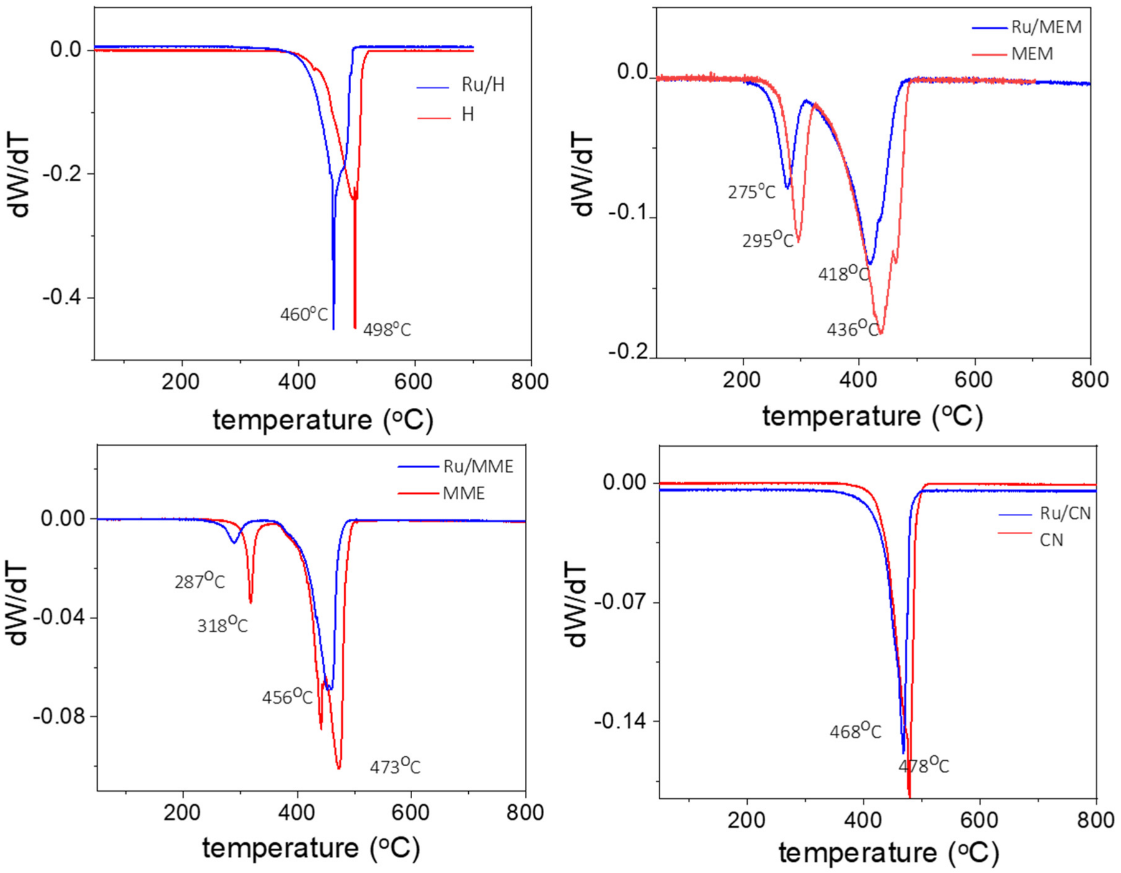

Figure S22. TGA analyses of ILs (red lines) and of RuNPs/ILs (blue lines).

6. XPS analysis of ILs and RuNPs/ILs

XPS analysis of ILs

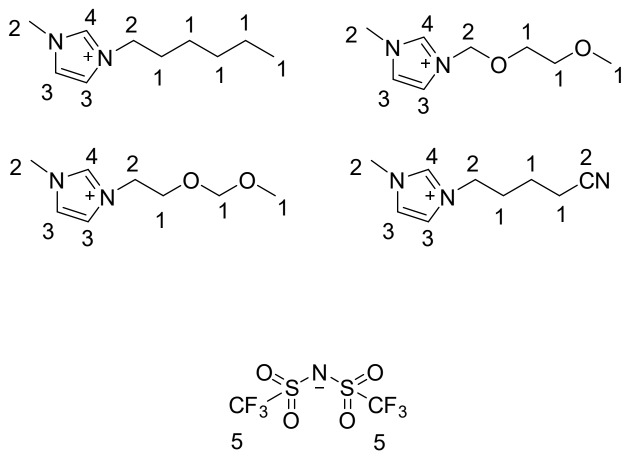

Figure S23. Chemical structures of the ILs with numbering of atoms.

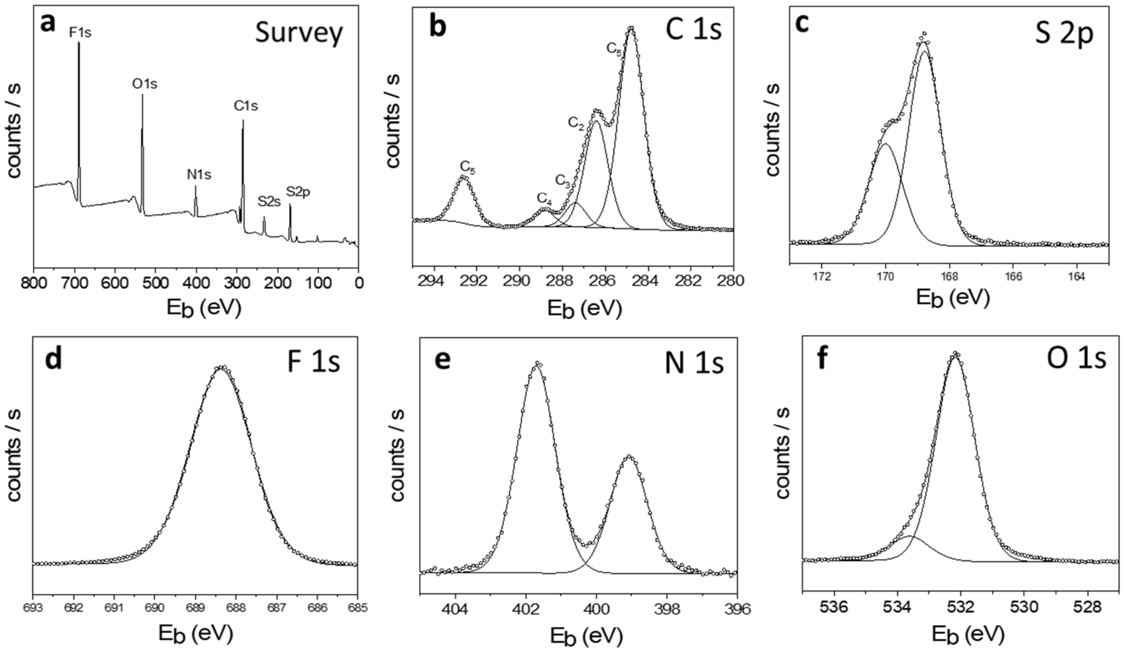

Figure S24. a) XPS survey spectrum of H and high-resolution scan spectra of b) C 1s, c) S 2p d) F 1s, e) N 1s, and f) O 1s.

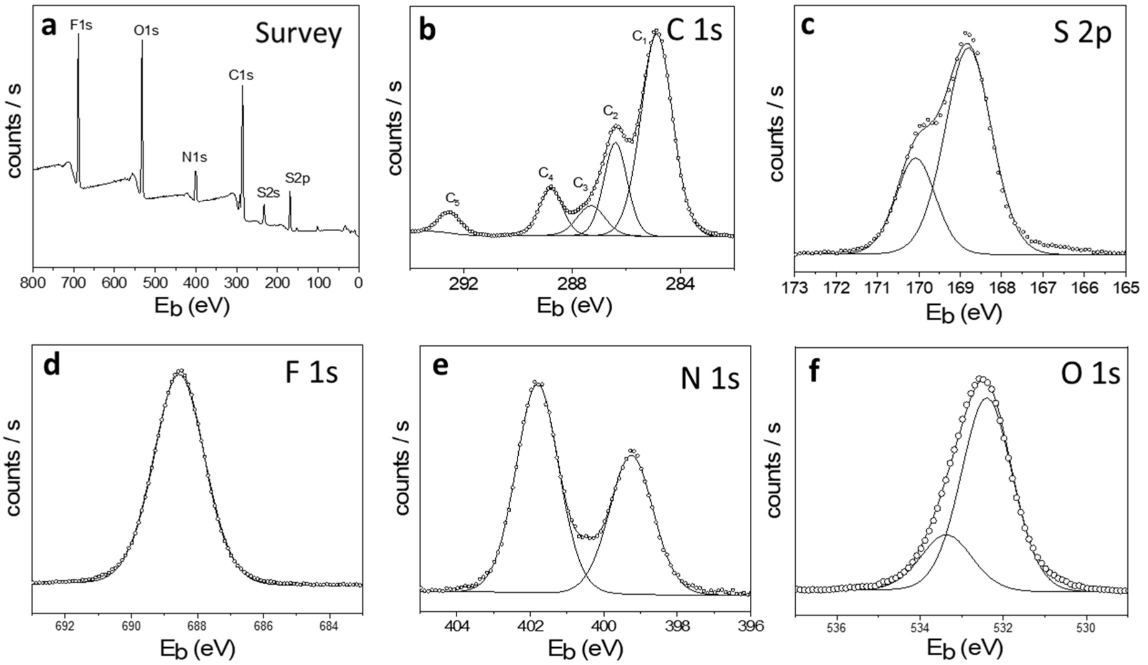

Figure S25. a) XPS survey spectrum of MEM and high-resolution scan spectra of b) C 1s, c) S 2p d) F 1s, e) N 1s, and f) O 1s.

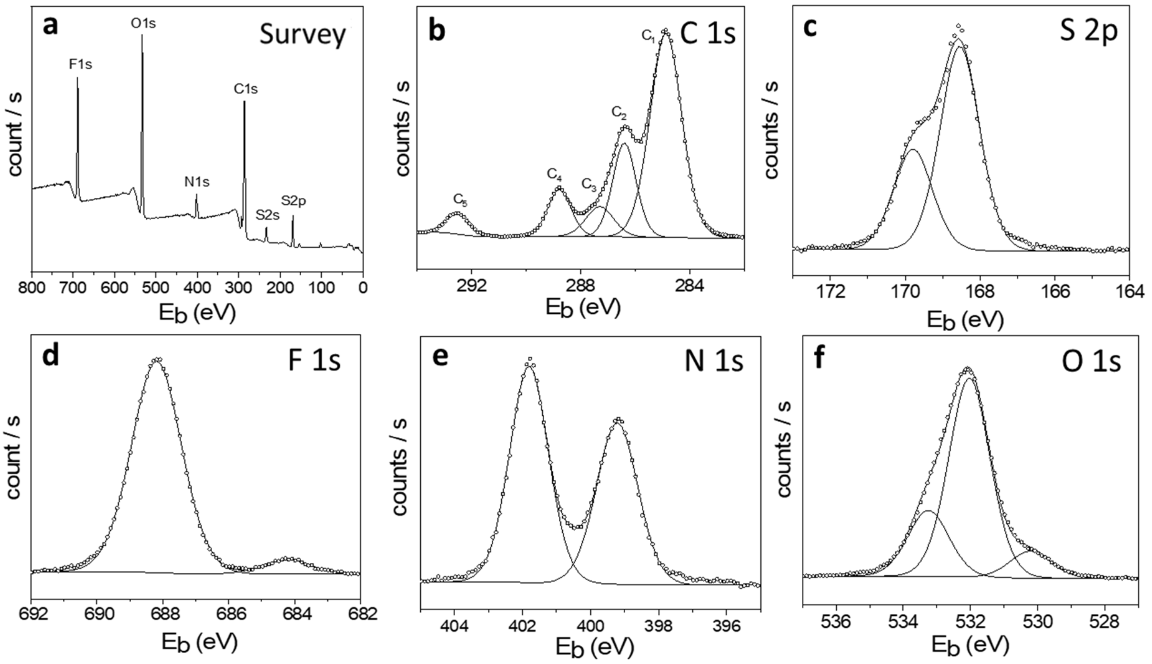

Figure S26. a) XPS survey spectrum of MME and high-resolution scan spectra of b) C 1s, c) S 2p d) F 1s, e) N 1s, and f) O 1s.

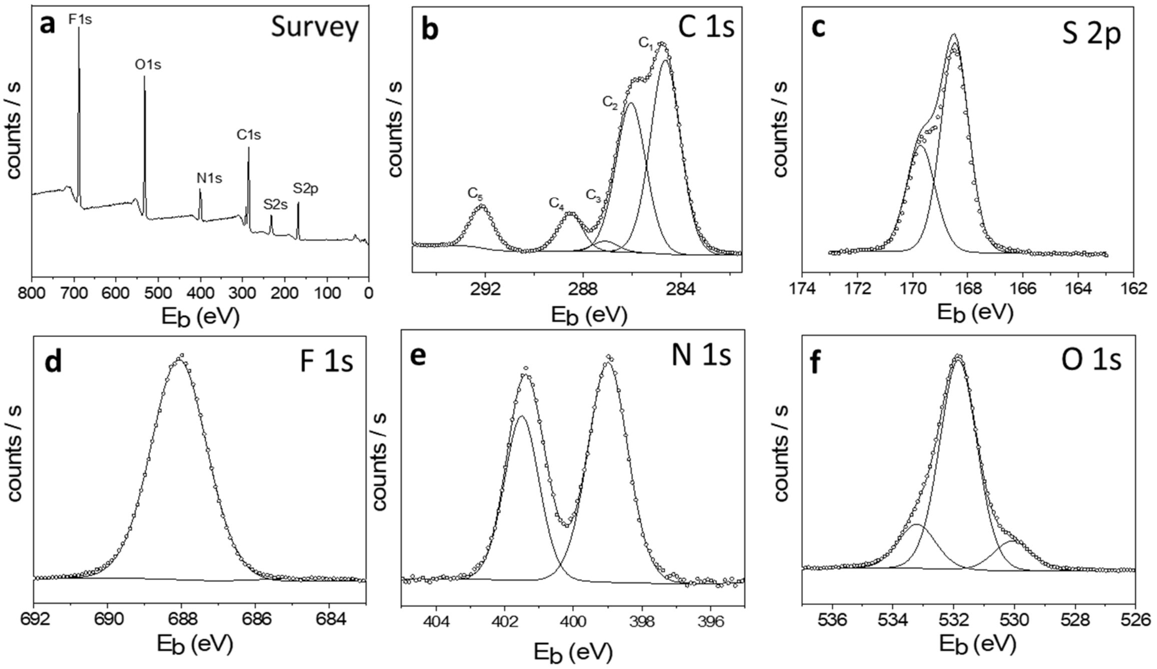

Figure S27. a) XPS survey spectrum of CN and high-resolution scan spectra of b) C 1s, c) S 2p d) F 1s, e) N 1s, and f) O 1s.

<u>XPS analysis of RuNPs/ILs</u>

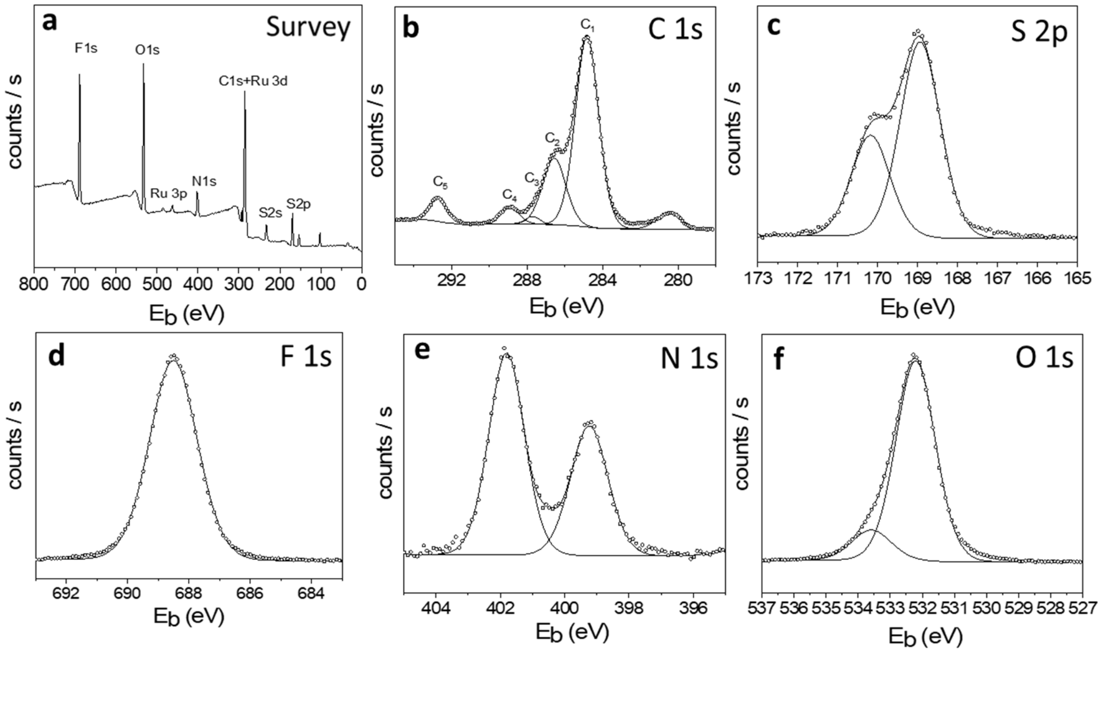

Figure S28. a) XPS survey spectrum of RuNPs/H and high-resolution scan spectra of b) C 1s, c) S 2p d) F 1s, e) N 1s, and f) O 1s.

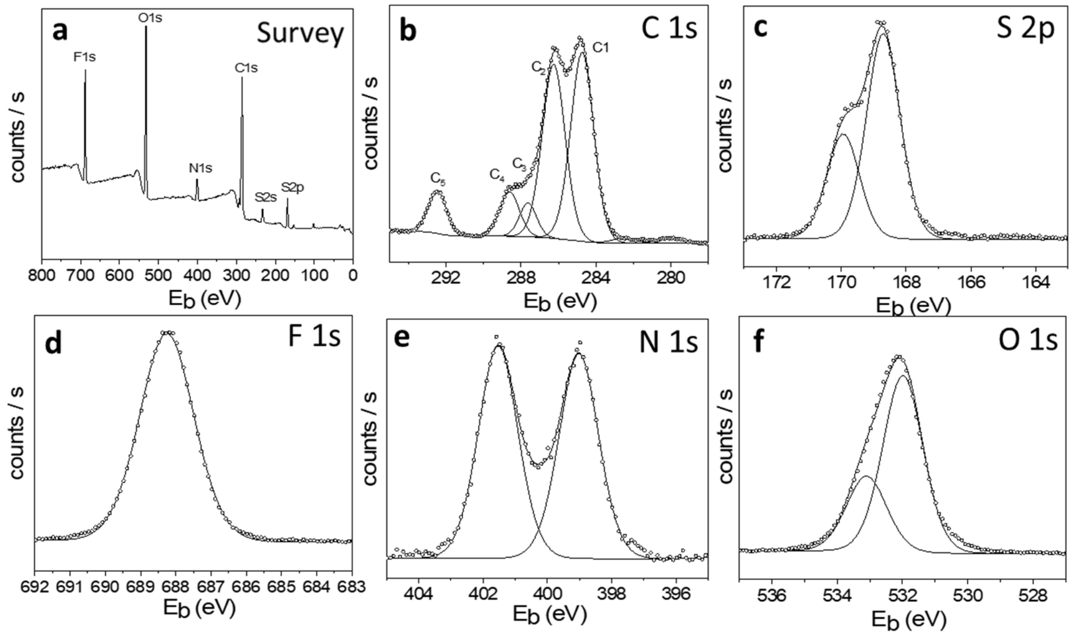

Figure S29. a) XPS survey spectrum of RuNPs/MEM and high-resolution scan spectra of b) C 1s, c) S 2p d) F 1s, e) N 1s, and f) O 1s.

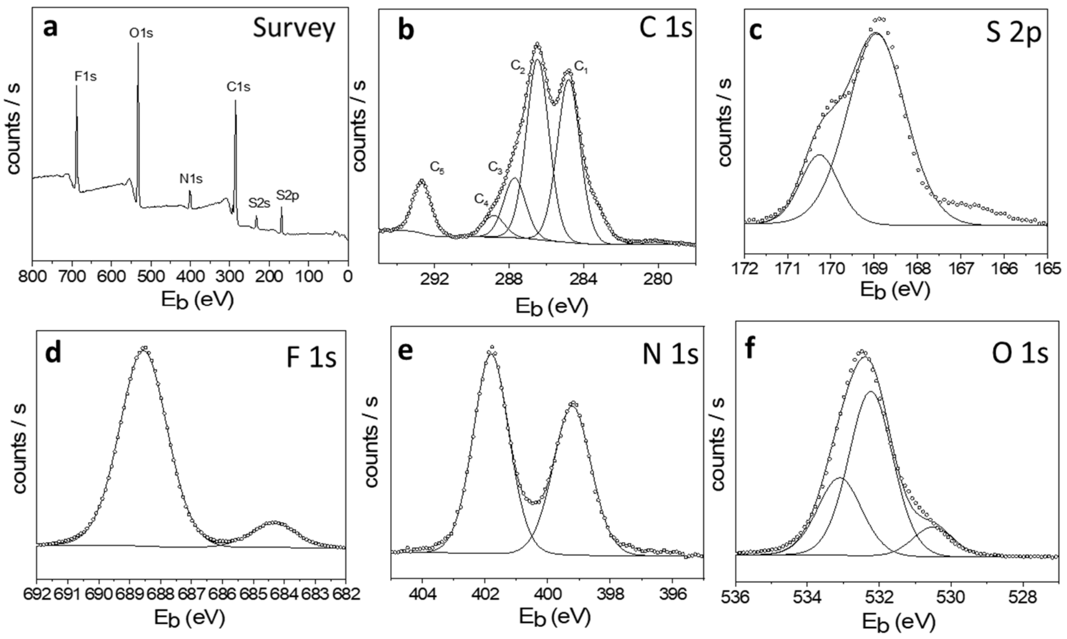

Figure S30. a) XPS survey spectrum of RuNPs/MME and high-resolution scan spectra of b) C 1s, c) S 2p d) F 1s, e) N 1s, and f) O 1s.

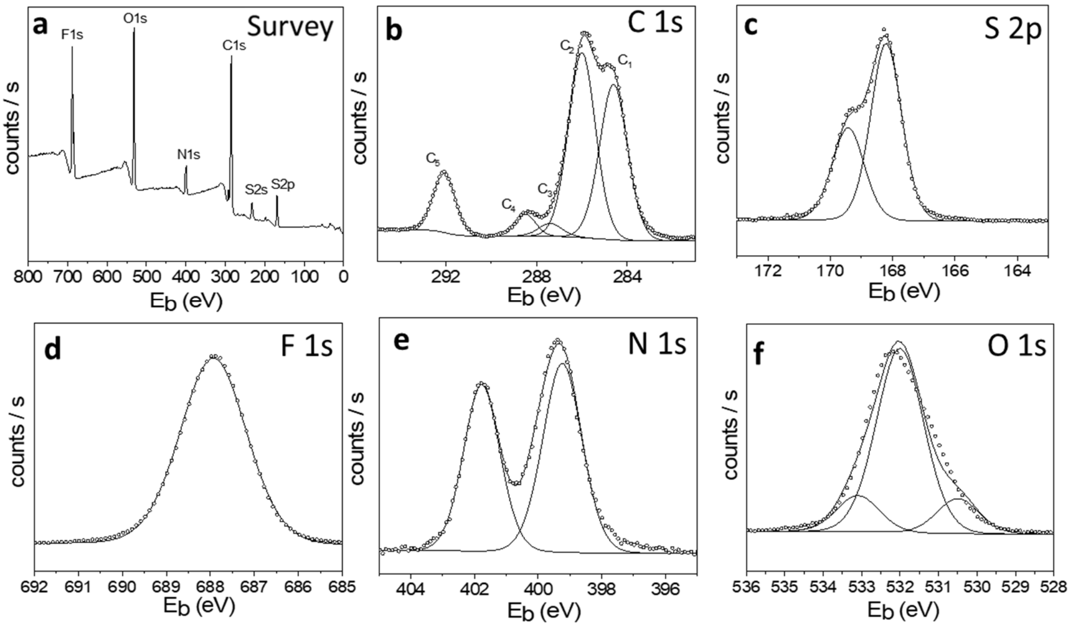

Figure S31. a) XPS survey spectrum of RuNPs/CN; and high-resolution scan spectra of b) C 1s, c) S 2p d) F 1s, e) N 1s, and f) O 1s.

<u>XPS analysis of RuNPs in the different ILs</u>

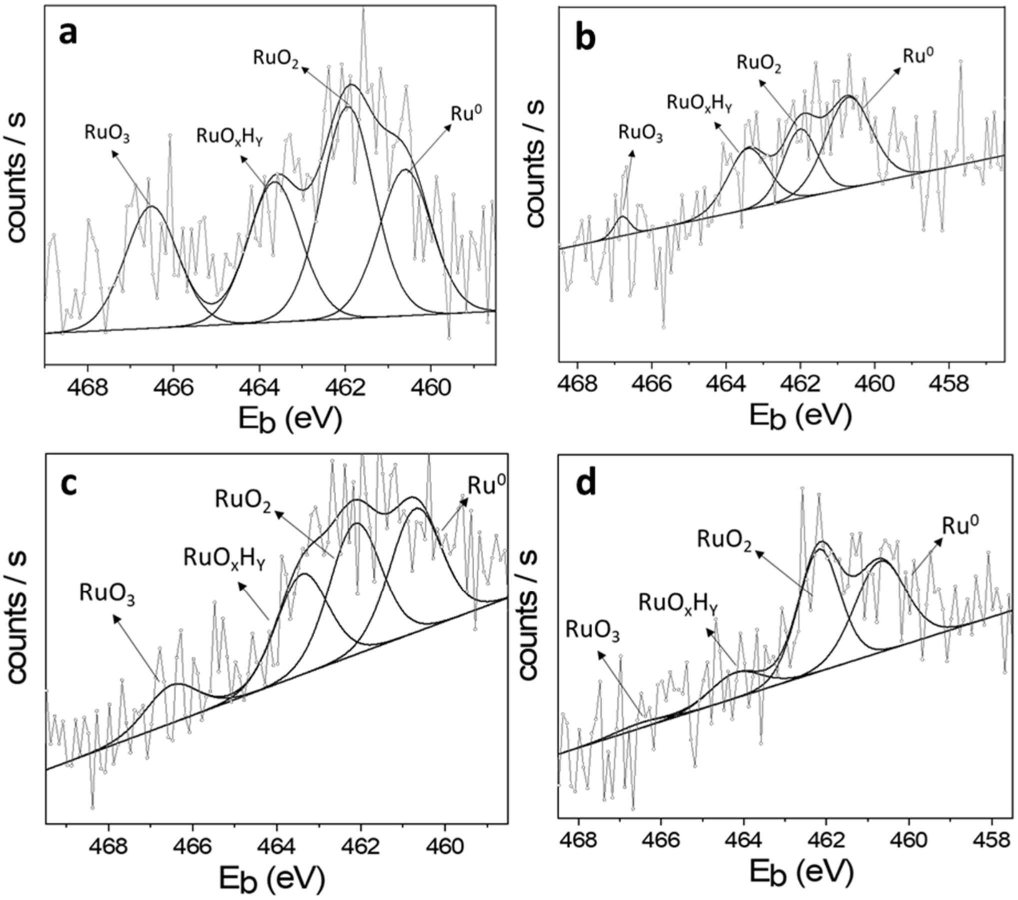

Figure S32. High-resolution XPS scan spectra of Ru 3p a) H, b) MEM, c) MME, and d) CN.

Table S1. Binding energies (eV) of C 1s for the carbon atoms in the ILs and RuNPs/ILs.

Atom[a]	Binding Energy IL (E_{IL})	Atomic ratio (%)	FWHM (eV)	Binding Energy RuNPs/ILs (E_{RuIL})	Atomic ratio (%)	FWHM (eV)	ΔE[b] (eV)
				H			
C_1	284.80	52.73	1.37	284.80	56.60	1.44	0.00
C_2	286.43	26.55	1.29	286.53	22.45	1.44	0.10
C_3	287.41	5.310	1.14	287.68	2.19	1.01	0.27
C_4	288.85	3.710	1.14	288.92	3.98	1.12	0.07
C_5	292.59	10.15	1.13	292.66	6.49	1.13	0.07
				MEM			
C_1	284.91	28.00	1.30	284.91	40.26	1.44	0.00
C_2	286.47	11.10	1.20	286.42	36.93	1.43	-0.05
C_3	287.70	3.10	1.20	287.79	5.36	1.06	0.09
C_4	288.57	5.00	1.00	288.77	8.05	1.22	0.20
C_5	292.70	2.10	1.00	292.62	7.13	1.13	-0.08
				MME			
C_1	284.90	28.0	1.30	284.90	32.78	1.44	0.00
C_2	286.40	11.10	1.20	286.46	35.95	1.43	0.06
C_3	287.50	3.10	1.20	287.79	11.51	1.38	0.29
C_4	288.8	5.00	1.00	288.81	3.99	1.33	0.01
C_5	292.5	2.10	1.00	292.66	8.42	1.16	0.16
				CN			
C_1	284.91	46.03	1.44	284.94	37.34	1.44	0.03
C_2	286.04	35.50	1.44	286.05	43.33	1.41	0.01
C_3	287.40	2.07	1.21	287.11	2.63	1.23	-0.29
C_4	288.50	7.25	1.18	288.53	3.93	1.06	0.03
C_5	292.07	7.83	1.14	292.14	11.38	1.14	0.07

[a] C_1-C_5 correspond to the carbon atoms in the ILs as numbered in Figure S32. [b] $\Delta E = E_{IL} - E_{RuIL}$.

Table S2: Binding energies (eV) of the chemical states of the atoms in the ILs and RuNPs/ILs.

Element	Binding Energy IL (E_{IL})	Atomic ratio (%)	FWHM (eV)	Binding Energy RuNPs/ILs (E_{RuIL})	Atomic ratio (%)	FWHM (eV)	ΔE (eV)
				H			
N 1s (anion)	399.08	35.00	1.25	399.24	39.18	1.37	0.16
N 1s (cation)	401.71	65.00	1.27	401.8	60.82	1.30	0.09
S $2p_{3/2}$	168.78	64.58	1.18	168.93	66.65	1.18	0.15
S $2p_{1/2}$	170	35.42	1.25	170.17	33.35	1.14	0.17
F 1s	688.38	100	1.79	688.5	100	1.83	0.12
O 1s (cation/anion)	532.17	89.39	1.44	532.21	86.9	1.44	0.04
$H_2O_{absorbed}$	533.62	10.61	1.44	533.59	13.10	1.44	0.03
Ru $3p_{3/2}$	-	-	-	460.90	30.49	1.44	-
				MEM			
N 1s (anion)	399.02	49.80	1.44	399.25	40.62	1.41	0.23
N 1s (cation)	401.53	50.21	1.44	401.81	58.91	1.36	0.28
S $2p_{3/2}$	168.7	66.84	1.22	168.81	71.69	1.33	0.11
S $2p_{1/2}$	169.94	33.16	1.19	170.08	28.31	1.12	0.14
F 1s	688.26	100	1.82	688.55	100	1.83	0.29
O 1s (cation/anion)	531.99	70.18	1.44	532.40	77.75	1.44	0.41
$H_2O_{absorbed}$	533.10	29.82	1.44	533.17	22.25	1.44	0.07
Ru $3p_{3/2}$	-	-	-	460.70	44.83	1.44	-
				MME			
N 1s (anion)	398.91	36.85	1.29	399.22	43.24	1.43	0.31
N 1s (cation)	401.52	61.42	1.32	401.81	56.76	1.34	0.29
S $2p_{3/2}$	168.54	67.40	1.24	168.94	80.65	1.57	0.4
S $2p_{1/2}$	169.79	32.60	1.22	170.26	19.35	1.04	0.47
F 1s (LiF)	684.24	6.03	1.70	684.36	10.69	1.75	0.12
F 1s (anion)	688.19	93.97	1.79	688.55	89.31	1.82	0.36
O 1s (CO_3^{2-})	530.22	9.15	1.44	530.20	10.24	1.33	0.02
O 1s (cation/anion)	532.03	68.25	1.44	532.23	61.06	1.44	0.2
$H_2O_{absorbed}$	533.25	22.60	1.44	533.10	28.70	1.44	0.15
Ru $3p_{3/2}$	-	-	-	460.70	31.42	1.44	-

CN

N 1s (anion)	398.99	59.91	1.37	399.23	54.34	1.44	0.24
N 1s (cation)	401.50	40.36	1.19	401.77	45.36	1.34	0.27
S $2p_{3/2}$	168.47	65.51	1.19	168.82	64.85	1.1	0.35
S $2p_{1/2}$	169.71	34.49	1.23	169.99	35.15	1.34	0.28
F 1s	687.93	97.39	1.77	688.06	97.86	1.78	0.13
O 1s (CO_3^{2-})	530.07	10.39	1.44	530.50	6.52	1.44	0.43
O 1s (cation/anion)	532.04	74.04	1.44	532.00	84.13	1.41	0.04
$H_2O_{absorbed}$	533.21	15.57	1.44	533.27	9.35	1.26	0.06
Ru $3p_{3/2}$	-	-	-	460.70	31.42	1.44	-

Each survey scan was performed six times, Ru $3p_{3/2}$ scans either 35 (H sample) or 150 (other ILs) times and all other elements scanned ten times. The software's automatic survey ID function was used to obtain the atomic surface concentrations from survey scans. “Smart” backgrounds were applied to all spectra except for Ru $3p_{3/2}$ for which the “Shirley” method was used. Background-corrected spectra were deconvoluted using Lorentzian/Gaussian (30/70%) curves for identification and quantification of chemical species.

7. Solubility of styrene (S) and ethylbenzene (EB) in ILs

Table S3. Solubility of S and EB in ILs.

IL	Solubility (mmol/mL)	
	S	EB
HM	5.42	6.46
MEM	2.66	2.41
MME	2.63	1.61
CN	2.14	1.42

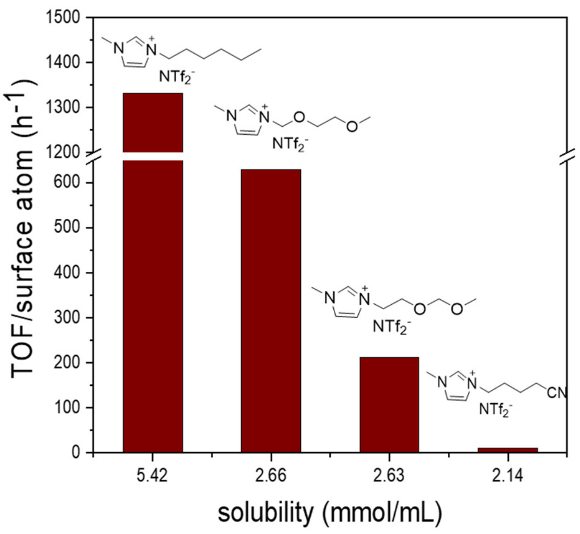

Figure S33. Solubility of S in the ILs (mmol/mL) vs TOF/(surface atom) add S in the x axis of the graph.

8. Hydrogenation catalysis with RuNPs/ILs

Results obtained with Ru/H

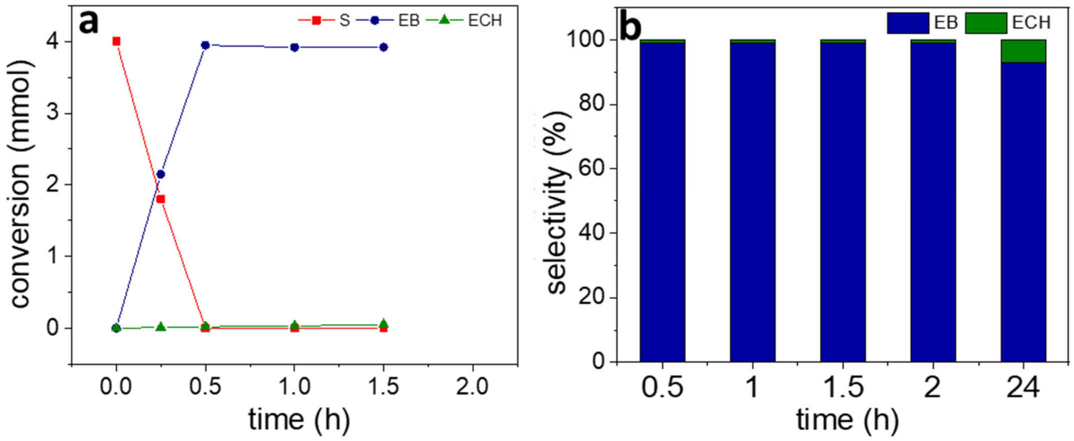

Figure S34. a) S conversion and B) selectivity into EB and ECH with time.

Results obtained with Ru/MEM

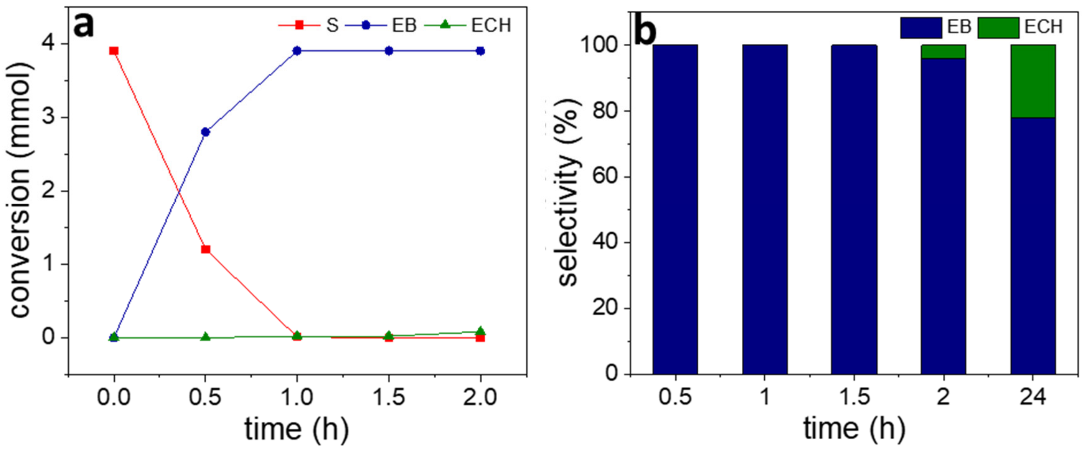

Figure S35. a) S conversion and b) selectivity into EB and ECH with time.

Results obtained with Ru/MME

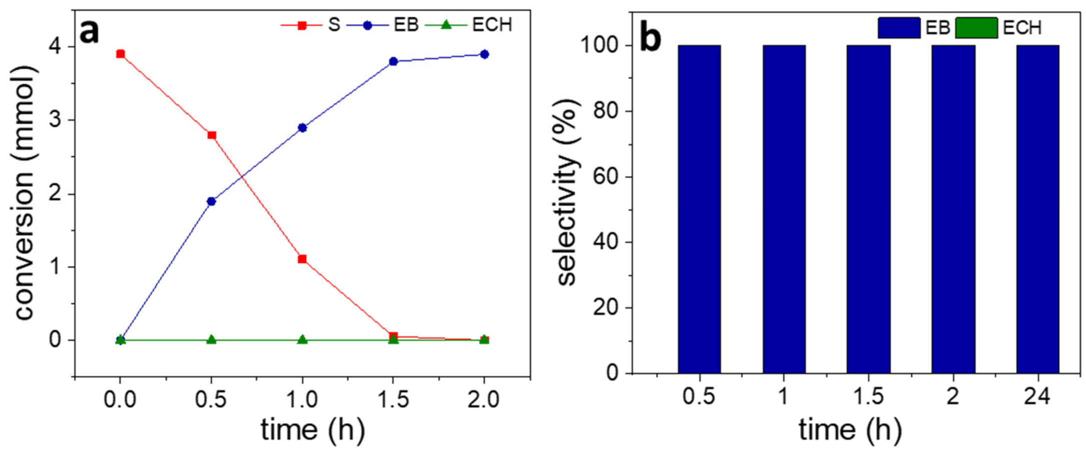

Figure S36. a) S conversion and b) selectivity into EB and ECH with time.

Results obtained with Ru/CN

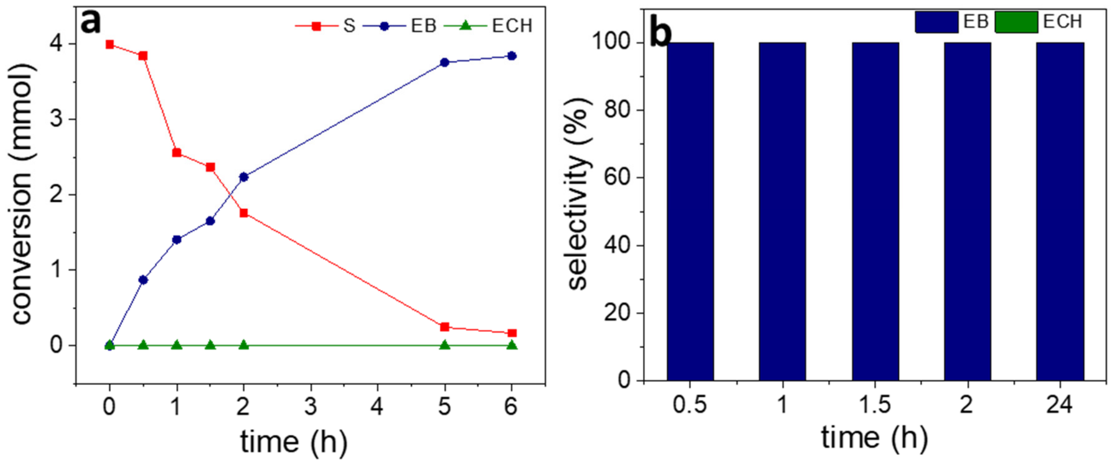

Figure S37. a) S conversion and b) selectivity into EB and ECH with time.

9. Calculation of TOFs

Table S4: Calculation of TOFs.

RuNPs/ILs	NP mean size (nm)[a]	Ru atoms in NP[b]	Ru atoms in core[c]	Ru atoms in outer layer[d]	Ratio of Ru atoms at NP surface[e]	TOF (h^{-1})[f]
Ru/H	1.5 ± 0.8	130	47	83	0.63	1,332
Ru/MEM	2.2 ± 0.3	409	214	195	0.48	629
Ru/MME	1.3 ± 0.1	84	25	59	0.70	212
Ru/CN	1.3 ± 0.2	84	25	59	0.70	10

[a] Mean size of Ru/IL NPs were determined from analysis of TEM images by considering at least 200 individual nanoparticles. Number of atoms in RuNPs were calculated as follows: the number of Ru atoms in *hcp* cell (N) is 6. The shell thickness is assumed as the half of the lattice constant, for Ru is 0.214 nm. The volume of Ru cell is 0.0817 nm^3. R_{np} represents the radius of NP. The volume of all Ru atoms in the outer layer of NP: $V_{shell} = V_{total} – V_{core} = 4/3\cdot\pi\cdot R_{np}^3 - 4/3\cdot\pi\cdot(R_{np}- R_{Ru})^3$, V_{total} means the volume of one RuNP, V_{core} corresponds to the volume of NP excluded the outer layer of atoms. [b] The total number of Ru atoms $N_{total} = N\cdot V_{total}/0.0817$. [c] The number of metal atoms in the core $N_{core} = N_{total} – N_{shell}$. [d] The numbers of metal atoms on the shell $N_{shell} = N\cdot V_{shell}/0.0817$. [e] The ratio of Ru atoms on the surface of NP = N_{shell}/N_{total}. [f] TOFs calculated at isoconversion (55%) with respect to the surface Ru atom ratio.

10. TEM analysis of RuNPs/ILs after five catalytic runs

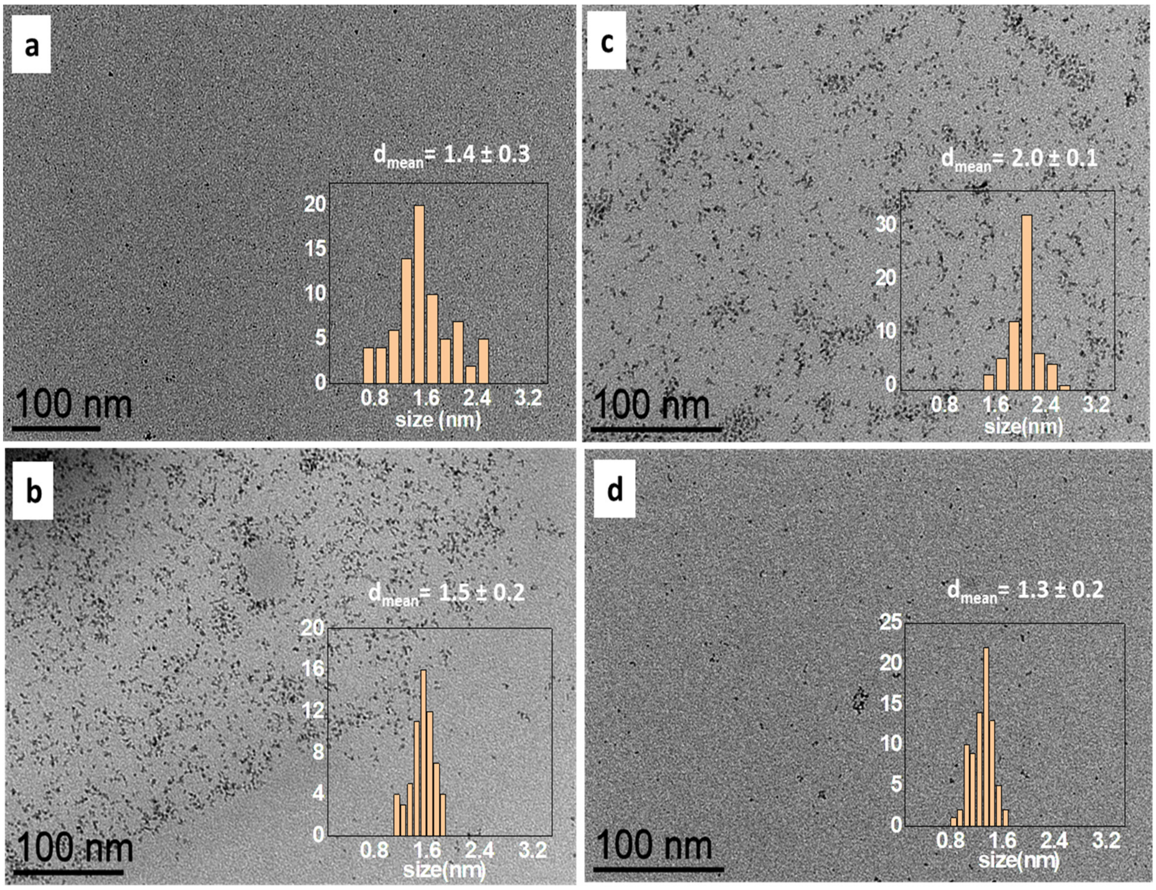

Figure S38. TEM images of the RuNPs/ILs systems a) Ru/H, b) Ru/MEM, c) Ru/MME, and d) Ru/CN after 5 catalytic runs with their corresponding size distribution.
